# Estimating physical activity from self-reported behaviours in large-scale population studies using network harmonisation: findings from UK Biobank and associations with disease outcomes

**DOI:** 10.1186/s12966-020-00937-4

**Published:** 2020-03-16

**Authors:** Matthew Pearce, Tessa Strain, Youngwon Kim, Stephen J. Sharp, Kate Westgate, Katrien Wijndaele, Tomas Gonzales, Nicholas J. Wareham, Søren Brage

**Affiliations:** 1 MRC Epidemiology Unit, University of Cambridge School of Clinical Medicine, Institute of Metabolic Science, Cambridge Biomedical Campus, Box 285, Cambridge, CB2 0QQ UK; 2 School of Public Health, The University of Hong Kong Li Ka Shing Faculty of Medicine, Room 301D 3/F, Jockey Club Building for Interdisciplinary Research, 5 Sassoon Road, Pokfulam, Hong Kong

**Keywords:** Accelerometer, Physical activity energy expenditure, Questionnaire, Calibration, Doubly labelled water

## Abstract

**Background:**

UK Biobank is a large prospective cohort study containing accelerometer-based physical activity data with strong validity collected from 100,000 participants approximately 5 years after baseline. In contrast, the main cohort has multiple self-reported physical behaviours from > 500,000 participants with longer follow-up time, offering several epidemiological advantages. However, questionnaire methods typically suffer from greater measurement error, and at present there is no tested method for combining these diverse self-reported data to more comprehensively assess the overall dose of physical activity. This study aimed to use the accelerometry sub-cohort to calibrate the self-reported behavioural variables to produce a harmonised estimate of physical activity energy expenditure, and subsequently examine its reliability, validity, and associations with disease outcomes.

**Methods:**

We calibrated 14 self-reported behavioural variables from the UK Biobank main cohort using the wrist accelerometry sub-cohort (*n* = 93,425), and used published equations to estimate physical activity energy expenditure (PAEE_SR_). For comparison, we estimated physical activity based on the scoring criteria of the International Physical Activity Questionnaire, and by summing variables for occupational and leisure-time physical activity with no calibration. Test-retest reliability was assessed using data from the UK Biobank repeat assessment (*n* = 18,905) collected a mean of 4.3 years after baseline. Validity was assessed in an independent validation study (*n* = 98) with estimates based on doubly labelled water (PAEE_DLW_). In the main UK Biobank cohort (*n* = 374,352), Cox regression was used to estimate associations between PAEE_SR_ and fatal and non-fatal outcomes including all-cause, cardiovascular diseases, respiratory diseases, and cancers.

**Results:**

PAEE_SR_ explained 27% variance in gold-standard PAEE_DLW_ estimates, with no mean bias. However, error was strongly correlated with PAEE_DLW_ (*r* = −.98; *p* < 0.001), and PAEE_SR_ had narrower range than the criterion. Test-retest reliability (Λ = .67) and relative validity (Spearman = .52) of PAEE_SR_ outperformed two common approaches for processing self-report data with no calibration. Predictive validity was demonstrated by associations with morbidity and mortality, e.g. 14% (95%CI: 11–17%) lower mortality for individuals meeting lower physical activity guidelines.

**Conclusions:**

The PAEE_SR_ variable has good reliability and validity for ranking individuals, with no mean bias but correlated error at individual-level. PAEE_SR_ outperformed uncalibrated estimates and showed stronger inverse associations with disease outcomes.

## Background

Higher levels of physical activity have been shown to be associated with a lower risk of morbidity and mortality [1], but accurately assessing the dose of physical activity in large population studies remains challenging. Most large cohort studies with long follow-up have utilised self-report questionnaires to assess physical activity. These methods typically have lower cost and higher feasibility than more objective methods but are prone to measurement error [2], and may not capture physical activity across all activity domains meaning the full dose is not characterised [3]. UK Biobank has shown that it is feasible to collect accelerometer-based physical activity data with strong validity [4] on a large scale (*n* > 100,000) [5]. Despite this, the main UK Biobank cohort is five times larger and has longer follow-up time to morbidity and mortality outcomes, which offers several epidemiological advantages compared to the more recent accelerometer sub-cohort. However, there is currently no tested method for estimating total volume of physical activity from the self-report information in UK Biobank collected at baseline.

The baseline questionnaire includes items adapted from the International Physical Activity Questionnaire (IPAQ) [6] and the Recent Physical Activity Questionnaire (RPAQ) [7, 8]. Responses could theoretically be processed separately using methods developed specifically for those two questionnaires, but using the totality of the available data should provide a more comprehensive estimate of the total dose, as they capture information about complimentary types, intensities and domains of activity. Previous work has shown how these self-reported behaviours relate to a summary of movement volume from 24-h wrist acceleration [9], and how wrist acceleration relates to physical activity energy expenditure (PAEE) as measured by the gold-standard method of doubly labelled water [4]. Despite the paucity of validation studies describing the direct relationship between these self-report data and those from the gold-standard method, it is possible to use network harmonisation [10] to combine the above strands of evidence to estimate PAEE; this would capitalise on the very large sample size of strand one and the more robust relationship between two objective measures in strand two, but the reliability and validity of this approach have not yet been tested in this context.

This study aimed to: 1) use the UK Biobank accelerometry sub-cohort to harmonise the self-reported behavioural variables and produce a summary estimate of PAEE; 2) examine test-retest reliability of this estimate using the UK Biobank repeat assessment sub-cohort; 3) assess validity of the PAEE estimate using values from a gold-standard doubly labelled water (DLW) based assessment in an independent validation study; 4) investigate associations of the PAEE estimate with morbidity and mortality in the main UK Biobank cohort.

## Methods

The following sections set out the collection and processing of relevant data in UK Biobank, the methods of the DLW validation study, and the statistical analyses.

### UK Biobank

#### Participants and study design

UK Biobank is an ongoing prospective cohort study of 502,625 adults aged 40–69 years residing within 25 miles of one of 22 assessment centres in England, Scotland, and Wales. Additional file 1: Figure S1 describes the exclusion criteria and sample sizes used in different components of the present study. Participants were identified from National Health Service general practitioner registries and invited to a baseline assessment between 2006 and 2010 [11]. A subsample of 20,346 participants attended a repeat assessment visit (2012–2013), and between 2013 and 2015 another partially overlapping subsample of 106,053 participated in a follow-up study during which they wore a wrist-mounted accelerometer for 7 days [5]. The UK Biobank study was approved by the North West Multicentre Research Ethics Committee and all participants provided written informed consent. Data for the current analysis were downloaded on 4th April 2019, containing information from 502,536 participants with baseline measures following withdrawals.

#### Self-reported behaviours

Physical activity, television viewing, computer use, and sleep were self-reported using a touch-screen questionnaire and responses were used to generate behavioural variables as previously described [9]. There are a total of 14 behavioural variables which are detailed in Supplementary Table S1; data for these were collected at baseline (2006–2010) and in a subsample during the repeat-assessment visit (2012–2013). IPAQ-based questions were used to derive minutes per day of moderate-to-vigorous physical activity (MVPA), as well as the IPAQ score in metabolic equivalent of task (MET) minutes/day for comparison [6] (Supplementary Table S2). Similarly, RPAQ-based questions were used to derive (minutes per day unless stated otherwise): walking for pleasure, strenuous sports, other exercises, light do-it-yourself (DIY), heavy DIY, heavy physical work, walking/standing work, sedentary work, getting about method (categorical: car or public transport, mixed use, walking or cycling), commuting method (categorical: car or public transport, mixed use, walking or cycling), television viewing (hours per day), computer use (hours per day). The questions are similar but not identical to those used in the original RPAQ [7]. Therefore, an alternative summary was computed for this instrument following the same scoring principles; this score in MET-minutes/day comprised the sum of leisure-time and occupational physical activity and is denoted LTPA+OPA in the present analysis (Supplementary Table S2). Sleep and nap time was categorised as: ≤ 5 h per day, 6 h per day, 7 h per day, 8 h per day, ≥ 9 h per day. As part of pilot testing, some participants completed a different baseline questionnaire to the rest of the main cohort; the data were incompatible and we therefore excluded these participants (*n* = 3797). We also removed participants for whom the sum of daily MVPA, television viewing, computer use and sleep was greater than 24 h (*n* = 4514). These variables were chosen as they should be mutually exclusive and thus used to detect generic misunderstanding of the behavioural questions.

#### Accelerometer sub-cohort

The collection and processing of the accelerometer data have been described in greater detail previously [5]. Between 2013 and 2015 invitations to participate in the accelerometer sub-cohort were sent to 236,519 participants who had provided a valid email address at recruitment. Consenting participants (*n* = 106,053) were sent an accelerometer (Axivity AX3, Newcastle upon Tyne, UK) initialised to capture three-dimensional acceleration at 100 Hz continuously for 7 days which they were asked to begin wearing immediately on their dominant wrist. Participants were asked to return the accelerometer via pre-paid envelope after the monitoring period. Euclidean norm minus one (ENMO) was calculated as the Euclidean norm (vector magnitude) of calibrated acceleration [12] in three axes minus one gravitational unit (1000 m-g) and negative values were truncated to zero [13]. Periods of ≥ 60 min during which the standard deviations (SD) of all three axes were < 13.0 m-g were identified as non-wear. Mean wrist ENMO in m-g was summarised across valid wear-time (data across the full 24 h spectrum and at least 72 h of wear in total) for each individual whilst minimising diurnal bias caused by non-wear [14].

#### Calibration models

In order to utilise the totality of the self-report information in UK Biobank, linear regression models were fitted to estimate the association between the 14 behavioural variables and movement volume (ENMO) using data from the accelerometry sub-cohort. Continuous self-report variables were natural log (log_*e*_(x + 1)) transformed (+ 1 due to zero values). Coefficients were mutually adjusted (i.e. entered in the same regression model) and derived separately for men and women. We also accounted for change in both age and season between baseline and the accelerometry assessment by adding delta terms to the regression models. Participants with < 72 h of wear time (*n* = 6310) or mean wrist ENMO ≥ 500 m-g (*n* = 4) were excluded. The standard error (SE) of each predicted PAEE was calculated using the variance-covariance matrix from the model and the values of each variable.

#### Prediction of PAEE from self-report (PAEE_SR_)

The sex-specific regression models developed in the accelerometry sub-cohort were used to predict mean wrist ENMO from self-report data in the main UK Biobank cohort. These predicted wrist ENMO values were then converted to PAEE_SR_ in kJ/day/kg using data from a similarly aged UK cohort [15] and a previously reported scaling equation for dominant wrist acceleration [4]. To assess reliability, this process was repeated for participants with complete self-report data collected during the repeat assessment visit (*n* = 18,905).

To propagate the uncertainty of the initial prediction of wrist ENMO and subsequent conversion to PAEE_SR_, predicted wrist ENMO values were resampled 100 times at random from normal distributions centered at each individual’s estimated wrist ENMO and its SE. In the same way, we sampled 100 beta and alpha coefficients used to convert wrist ENMO to PAEE_SR_. Wrist ENMO was then converted to PAEE_SR_ using the 100 sets of sampled values and coefficients. The mean and SD of the 100 predictions for each individual were used as the point estimate of PAEE_SR_ and its SE, respectively.

#### Outcome assessment for survival analyses

Vital status and primary or secondary diagnoses of hospital episodes of participants were established by linkage to national death registry data obtained from the Health and Social Care Information Centre for England and Wales and the Information Services Department for Scotland [11]. Censoring dates were 31st January 2018 in England and Wales, and 30th November 2016 in Scotland. International Classification of Diseases 10th edition codes were used to define disease outcomes as shown in Supplementary Table S3. Non-fatal outcomes were hospital episodes of heart failure, stroke, ischaemic heart disease, atrial fibrillation, all cardiovascular disease, chronic obstructive pulmonary disease, all respiratory disease, cancers including breast, prostate, endometrial, lung, colon, oesophageal, liver, gastric cardia, myeloid leukaemia, myeloma, rectum, bladder, malignant melanoma, and all cancer. Selection of site-specific cancer outcomes was based upon a previous review [16] and at least 25 events in the follow-up period. Fatal outcomes were all-cause mortality, cardiovascular disease mortality, respiratory disease mortality, and cancer mortality.

#### Covariate assessment for survival analyses

Demographic, lifestyle, and clinical variables were assessed at baseline by the aforementioned touch-screen questionnaire, verbal interview, or physical measurement. The following variables were considered as potential confounders of the relationship between PAEE_SR_ and all-cause mortality: age, sex, ethnicity (white/non-white), Townsend deprivation index, highest educational level (degree or above/any other qualification/no qualification), employment status (unemployed/in paid or self-employment), alcohol consumption (never/previous/current), smoking (never/previous/current), salt added to food (never/sometimes), oily fish intake (never/sometimes), fruit and vegetable intake (score from 0 to 4), processed and red meat intake (average weekly frequency in days per week), body mass index (BMI) in three categories (< 25, 25–30, ≥ 30 kg•m^− 2^), parental cancer history including history of bowel, lung, maternal breast cancer, or paternal prostate cancer (yes/no), parental history of heart disease, stroke, hypertension or diabetes (yes/no), use of blood pressure medication (yes/no), use of cholesterol lowering medication (yes/no), doctor-diagnosed diabetes or treatment with insulin (yes/no), doctor-diagnosed coronary heart disease, stroke or cancer (yes/no).

### DLW validation study

The validity of PAEE_SR_ values was assessed using DLW-based PAEE values (PAEE_DLW_) in an independent validation study, details of which have previously been reported [4]. Participants were 100 adults aged 40–70 years recruited from the Fenland Study [17, 18] and invited to two assessment visits separated by 9–14 days for gold-standard assessment of total energy expenditure [19–30]. Resting energy expenditure and diet-induced thermogenesis values were subtracted from total energy expenditure and divided by body mass yielding an estimate of total daily PAEE_DLW_ in kJ/day/kg. Participants also answered the UK Biobank questions needed to generate PAEE_SR_ using the calibration model described above, although data were incomplete for some (*n* = 2). Ethical approval for this study was obtained from Cambridge University Human Biology Research Ethics Committee (Ref: HBREC/2015.16). All participants provided written informed consent.

### Statistical analyses

#### Test-retest reliability of behavioural variables, PAEE_SR_, IPAQ, and LTPA+OPA

Test-retest reliability (repeatability) of the 14 behavioural variables as well as the PAEE_SR_, IPAQ, and LTPA+OPA summary scores was examined by regression of the repeat assessment measures (2012–2013) on baseline measures (2006–2010) yielding lambda coefficients [31] and their standard errors, while (weighted) Cohen’s kappa coefficients [32] were calculated for ordinal variables.

#### Validity of PAEE_SR_, IPAQ, and LTPA+OPA

Absolute validity (agreement) of the PAEE_SR_ values was assessed by calculating the mean bias and 95% limits of agreement [33] compared with PAEE_DLW._ We used PAEE_DLW_ as the criterion in the main analysis rather than the average between PAEE_SR_ and PAEE_DLW_, which has been recommended [34]. However, error in PAEE_DLW_ is very low compared to self-report, meaning PAEE_DLW_ is likely to be closer to the latent ‘true’ level of the exposure. The plot of PAEE_SR_ vs the average of PAEE_SR_ and PAEE_DLW_ was conducted as a sensitivity analysis. Precision was assessed by calculating root mean square error (RMSE), i.e. the square-root of the mean squared differences. Individual differences between PAEE_SR_ and PAEE_DLW_ were examined visually across the measurement range of the criterion. The association between each of PAEE_SR_, IPAQ, and LTPA + OPA with PAEE_DLW_ was modelled using linear regression. The relative validity (similar ranking of individuals) of the three summary scores was examined with Spearman’s rank-order correlation using PAEE_DLW_.

#### Survival analyses

In the main UK Biobank cohort, Cox regression with age as the underlying timescale was used to estimate associations between PAEE_SR_ and each of the fatal and non-fatal outcomes, adjusted for all covariates listed above, and in a separate model omitting BMI. Hazard ratios were presented per 5 kJ/day/kg of PAEE as this is approximately equivalent to the lower World Health Organization guideline of 150 min of moderate intensity activity per week [35]. Models were weighted using the inverse of the individual-level SE; weights were normalised such that the sum of weights equalled the analytical sample size. Individuals with missing exposure data (*n* = 20,133) or covariate data (*n* = 19,778) were excluded for the survival analyses, as were individuals with pre-baseline hospital episodes of ischaemic heart disease, stroke, respiratory disease or cancer as defined above (*n* = 55,574), and those with only self-reported doctor-diagnosed ischaemic heart disease, stroke, or cancer (*n* = 23,402). Finally, we excluded participants experiencing events in the first 2 years of follow-up (*n* = 986 for mortality; range 22 to 24,084 for non-fatal outcomes), meaning the final analysis sample for mortality analyses included 374,352 participants, with fewer for analyses of non-fatal outcomes. Breast and prostate cancer analyses were conducted in women only and men only, respectively.

For fatal outcomes, we compared the associations of each of the three summary scores (PAEE_SR_, IPAQ, and LTPA+OPA) using the modelling approach described above, and presented hazard ratios per 1 SD increment of each exposure. We also repeated this adding sleep as a covariate in the Cox regression model when using IPAQ and LTPA+OPA. In sensitivity analyses, hazard ratios were also estimated by quartile of PAEE_SR_ using all covariates, and in a separate model omitting BMI. We also replicated the main analysis described above in only those participants reporting pre-baseline disease and who did not die within 2 years of follow-up (*n* = 77,843). In addition, the associations between PAEE_SR_ and each of the disease outcomes were assessed using cubic spline regression models (3 knots) using all the covariates. For this analysis, we used a reference PAEE_SR_ level of a hypothetical man or woman reporting: no leisure-time physical activity, 8 hours per day of sedentary occupation, 2 hours per day of television viewing, 2 hours per day of computer use, motorised transport for commuting and getting about, and sleeping for ≥ 9 h per day. All analyses were conducted using STATA/SE 14.2 (StataCorp, TX, USA).

## Results

Baseline characteristics of participants from the studies included in analyses are shown in Table 1. Participants in the DLW validation study were, on average, 2 years younger and more active than those in UK Biobank. Following exclusions, 52,507 women and 41,918 men were included in the two separate regression analyses to predict wrist movement from self-report data. The resulting models explained 14 and 17% of variance in mean wrist ENMO (m-g) in women and men respectively. The sex-specific coefficients for the 14 behavioural variables are shown in Additional file 1: Table S4.

**Table 1:** Characteristics of participants in UK Biobank and the DLW validation study

	UK Biobank		Independent DLW validation study
	Main cohort	Accelerometer sub-cohort	
Analysis sample (n)	374,352	93,425	98
Age at baseline (years)	56 (8)	56 (8)	54 (7)
Age at postal follow-up (years)	–	62 (8)	–
Proportion of women	56%	56%	50%
Weight (kg)	78 (16)	77 (15)	77 (14)
Body mass index (kg/m^2^)	27 (5)	27 (5)	27 (3)
Minutes per day of:			
Heavy physical work	21 (51)	16 (43)	38 (65)
Walking/standing work	56 (91)	48 (84)	91 (107)
Sedentary work	110 (143)	122 (146)	144 (153)
MVPA	90 (110)	85 (99)	124 (125)
Walking for pleasure	14 (22)	15 (22)	16 (27)
Strenuous sports	2 (10)	3 (10)	4 (12)
Other exercises	9 (17)	10 (17)	12 (16)
Light DIY	10 (24)	11 (24)	11 (28)
Heavy DIY	6 (19)	6 (18)	7 (24)
Hours per day of:			
Television viewing	3 (2)	2 (1)	2 (1)
Computer use	1 (1)	1 (1)	2 (2)
Getting about method:			
Car or public transportation	48%	44%	41%
Mixed use	43%	47%	32%
Walking or cycling	9%	9%	27%
Commuting method:			
Car or public transportation	87%	85%	59%
Mixed use	8%	10%	19%
Walking or cycling	5%	5%	22%
Hours per day of sleep:			
≤ 5.0	5%	4%	0%
6.0	19%	18%	4%
7.0	40%	43%	32%
8.0	29%	29%	49%
≥ 9.0	7%	6%	15%
PAEE_SR_ (kJ/day/kg)	47 (4)	48 (4)	49 (4)
IPAQ scoring (MET-minutes/day)	373 (458)	357 (412)	509 (468)
LTPA+OPA scoring (MET-minutes/day)	380 (425)	349 (383)	572 (560)

*DIY* do-it-yourself, *DLW* doubly labelled water, *IPAQ* International Physical Activity Questionnaire, *LTPA+OPA* leisure-time and occupational physical activity, *MET* metabolic equivalent of task, *MVPA* Moderate-to-vigorous physical activity, *PAEE*_*SR*_ physical activity energy expenditure predicted from self-report

Values are means (standard deviations) unless otherwise stated

### Test-retest reliability of behavioural variables, PAEE_SR_, IPAQ, and LTPA+OPA scores

The mean (SD) time between baseline (2006–2010) and repeat assessment (2012–2013) was 4.3 (0.9) years. Table 2 summarises self-reported behaviours at both time points: the largest change in reported behaviours between baseline and repeat assessment was for occupational variables, all of which decreased in duration. Test-retest reliability was higher for PAEE_SR_ than for the IPAQ or LTPA+OPA scores of MET-minutes per day.

**Table 2:** Reliability of self-reported behaviours using baseline and repeat assessment in UK Biobank (*n* = 18,905)

	Baseline	Repeat	Lambda/kappa (SE)
Years between baseline and repeat:			
< 3		11%	
≥ 3 to < 4		24%	
≥ 4 to < 5		43%	
≥ 5 to < 6		21%	
≥ 6		1%	
Minutes per day of:			
Heavy physical work	16 (43)	11 (36)	Λ = 0.527 (0.005)
Walking/standing work	47 (82)	31 (70)	Λ = 0.482 (0.005)
Sedentary work	112 (143)	76 (128)	Λ = 0.609 (0.005)
MVPA	83 (99)	81 (94)	Λ = 0.479 (0.006)
Walking for pleasure	15 (22)	16 (24)	Λ = 0.520 (0.007)
Strenuous sports	3 (10)	2 (10)	Λ = 0.453 (0.006)
Other exercises	10 (18)	10 (17)	Λ = 0.456 (0.006)
Light DIY	11 (26)	10 (23)	Λ = 0.256 (0.006)
Heavy DIY	7 (19)	6 (17)	Λ = 0.273 (0.006)
Hours per day of:			
Television viewing	3 (1)	3 (2)	Λ = 0.821 (0.005)
Computer use	1 (1)	1 (1)	Λ = 0.547 (0.007)
Getting about method:			Κ = 0.324 (0.006)
Car or public transportation	49%	48%	
Mixed use	44%	45%	
Walking or cycling	7%	7%	
Commuting method:			Κ = 0.487 (0.006)
Car or public transportation	90%	93%	
Mixed use	7%	5%	
Walking or cycling	3%	2%	
Hours per day of sleep:			Κ = 0.500 (0.005)
≤ 5.0	3%	4%	
6.0	18%	17%	
7.0	42%	40%	
8.0	30%	31%	
≥ 9.0	7%	8%	
PAEE_SR_ (kJ/day/kg)	46 (4)	47 (4)	Λ = 0.671 (0.005)
IPAQ scoring (MET-minutes/day)	345 (411)	342 (395)	Λ = 0.489 (0.006)
LTPA+OPA scoring (MET-minutes/day)	349 (382)	281 (337)	Λ = 0.552 (0.005)

*DIY* do-it-yourself, *IPAQ* International Physical Activity Questionnaire, *LTPA+OPA* leisure-time and occupational physical activity, *MET* metabolic equivalent of task, *MVPA* moderate-to-vigorous physical activity, *PAEE*_*SR*_ physical activity energy expenditure predicted from self-report, *SE* standard error, Λ Lambda coefficient, Κ weighted kappa coefficient

Values are mean (standard deviation) unless otherwise stated

### Validity of PAEE_SR_, IPAQ, and LTPA+OPA scores

**Fig. 1:**
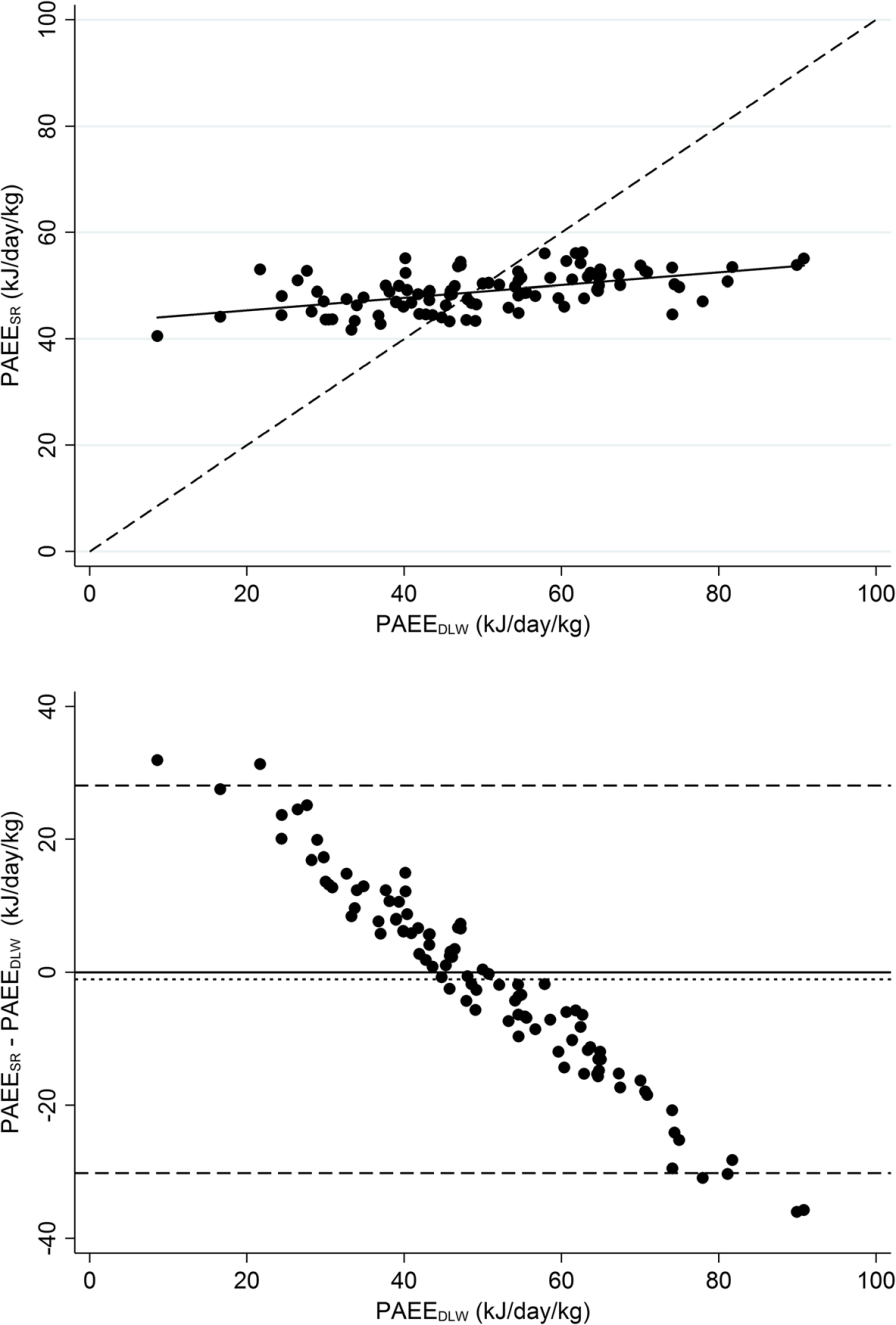
Validity of physical activity energy expenditure predicted from self-report (PAEE_SR_) vs. doubly labelled water based PAEE (PAEE_DLW_). Upper panel shows scatter plot with line of unity (dashed) and regression line (solid); lower panel shows differences between physical activity energy expenditure predicted from self-report (PAEE_SR_) and PAEE_DLW_, plotted against PAEE_DLW_. Reference lines indicate mean difference (dotted) and 95% limits of agreement (dashed). *n* = 98

Self-report data were complete for 98 out of 100 participants in the DLW validation study. Figure 1 shows PAEE_SR_ minus PAEE_DLW_ plotted against PAEE_DLW_. PAEE_DLW_ mean (SD) was 50.0 (16.1) kJ/day/kg compared with 48.9 (3.7) kJ/day/kg for PAEE_SR_. The mean bias was − 1.1 (95%CI: − 4.0 to 1.8 kJ/day/kg), or − 2% of the criterion mean, and the limits of agreement were − 30.2 to 28.1 kJ/day/kg (±58%). The RMSE was 14.5 kJ/day/kg, or 29% of the criterion mean. Error of PAEE_SR_ was strongly correlated with PAEE_DLW_ (*r* = −.98; *p* < 0.001); PAEE_SR_ was an overestimate for less active individuals and an underestimate for the more active. Plotting error of PAEE_SR_ vs the average of PAEE_SR_ and PAEE_DLW_ showed a similar proportional bias (*r* = −.93; *p* < 0.001, Supplemental Fig. S2). The range of PAEE_SR_ (40.5 to 56.2 kJ/day/kg) was 81% narrower than PAEE_DLW_ (9 to 91 kJ/day/kg). Spearman correlation between PAEE_SR_ and PAEE_DLW_ was *r*_s_ = .52 (*p* < 0.001), while for IPAQ and LTPA+OPA, Spearman correlations with PAEE_DLW_ were *r*_s_ = .23 (*p* = 0.022) and *r*_s_ = .41 (*p* < 0.001), respectively. PAEE_SR_ explained 27% of variance in PAEE_DLW_ with a large negative intercept (Fig. 1). By comparison, IPAQ and LTPA+OPA scores explained 5 and 8%, respectively.

### Survival analyses

**Fig. 2:**
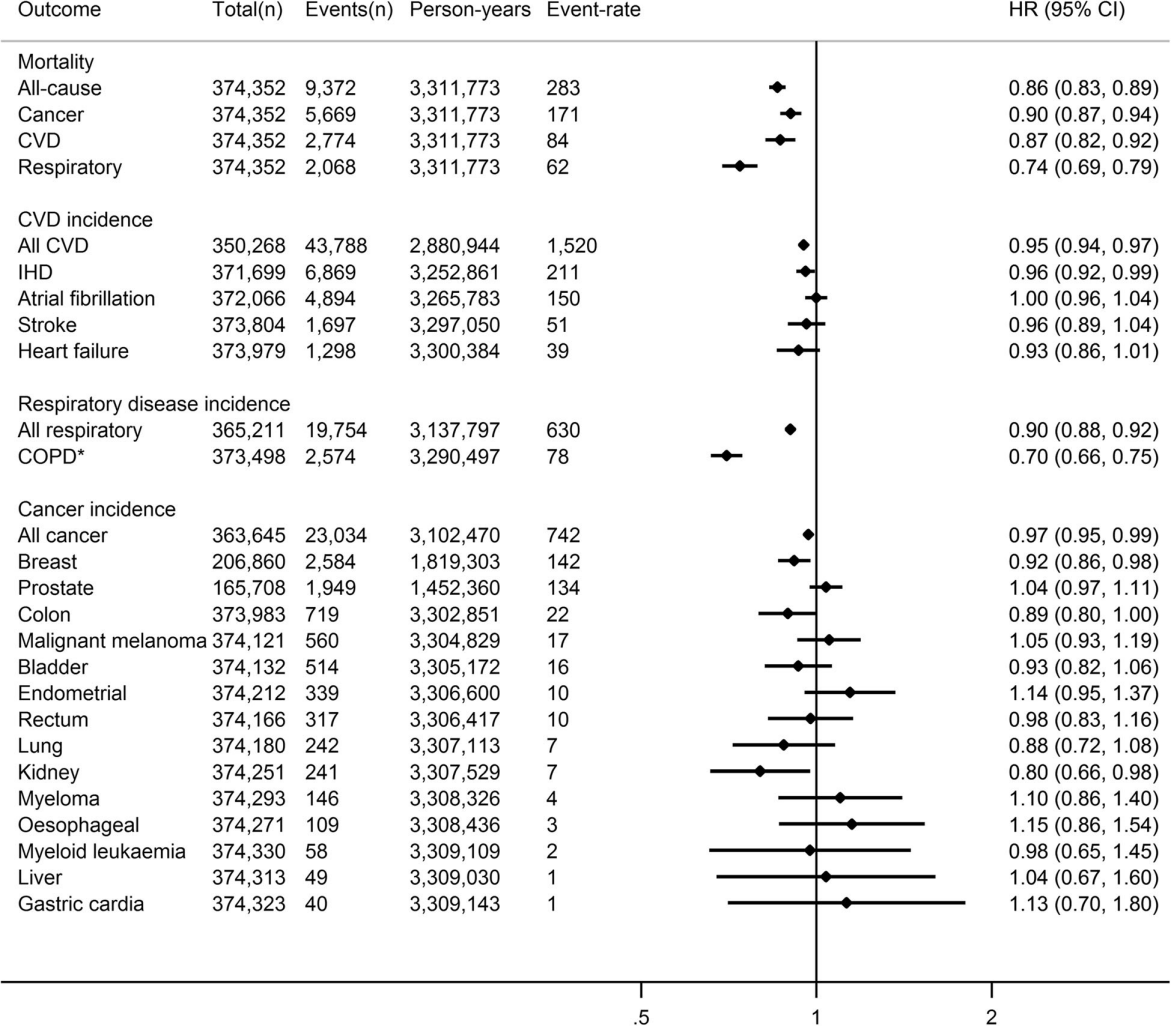
Hazard ratio (HR) and 95% confidence interval (CI) for linear associations of physical activity energy expenditure predicted from self-report (PAEE_SR_, per 5 kJ/day/kg increments) with fatal and non-fatal outcomes in UK Biobank. Event-rate per 100,000 person years. Adjusted for age (as timescale), sex, ethnicity, Townsend deprivation index (baseline hazard stratification), highest educational level, employment status, alcohol drinking status (baseline hazard stratification), smoking status, salt added to food, oily fish intake, fruit and vegetable intake, processed and red meat intake, body mass index, parental history of cancer, parental history of [heart disease, stroke, hypertension or diabetes], use of blood pressure medication, use of cholesterol lowering medication, doctor-diagnosed diabetes or treatment with insulin. *COPD*  chronic obstructive pulmonary disease; *CVD*  cardiovascular disease; *IHD*  ischaemic heart disease. *COPD incidence likely only represents the most severe cases as only approximately 25% of COPD cases are picked up in Hospital Episode Statistics data, compared to national surveys [36]

**Fig. 3:**
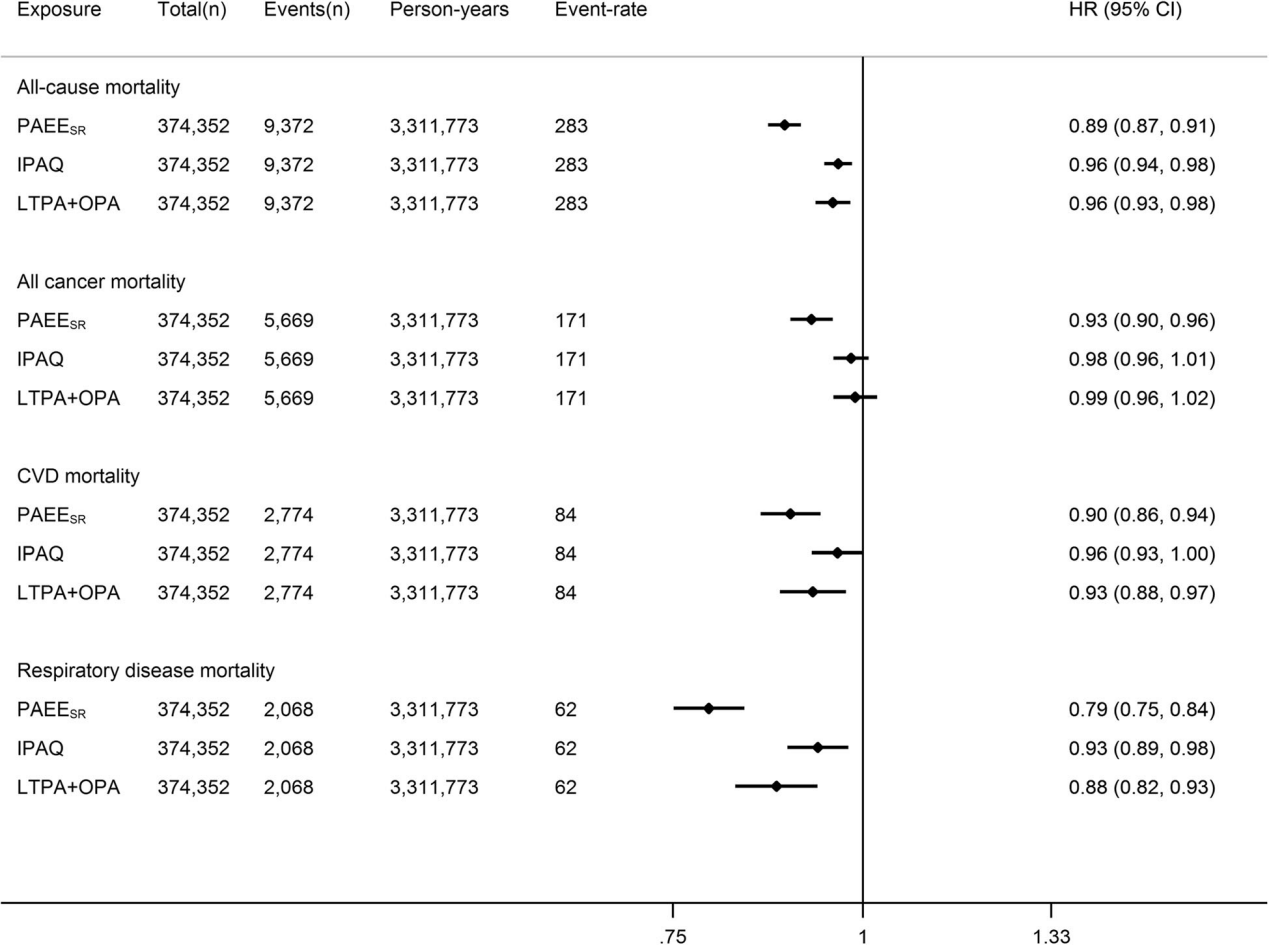
Hazard ratio (HR) and 95% confidence interval (CI) for linear associations between physical activity volume and mortality in UK Biobank. Physical activity volume is derived using three assessment methods: physical activity energy expenditure predicted from self-report (PAEE_SR_), International Physical Activity Questionnaire (IPAQ) scoring of MET-minutes/day, and sum of leisure-time physical activity and occupational physical activity MET-minutes/day (LTPA+OPA). All HRs per 1 standard deviation increment of exposure. Event-rate per 100,000 person years. Adjusted for age (as timescale), sex, ethnicity, Townsend deprivation index (baseline hazard stratification), highest educational level, employment status, alcohol drinking status (baseline hazard stratification), smoking status, salt added to food, oily fish intake, fruit and vegetable intake, processed and red meat intake, body mass index, parental history of cancer, parental history of [heart disease, stroke, hypertension or diabetes], use of blood pressure medication, use of cholesterol lowering medication, doctor-diagnosed diabetes or treatment with insulin. *CVD* cardiovascular disease, *MET* metabolic equivalent of task

During a median (interquartile range) 8.9 (8.3–9.5) years of follow-up (3,311,773 person-years), 9372 participants died. Each 5 kJ/day/kg of PAEE_SR_ (equivalent to meeting the lower activity recommendations) was associated with an approximate 14% lower hazard of all-cause mortality (Fig. 2). Incidence of non-fatal respiratory disease (but severe enough to require hospital admission) was more strongly associated with PAEE_SR_ than non-fatal cardiovascular disease or cancer incidence. Amongst site-specific cancers, PAEE_SR_ was only associated with non-fatal breast and kidney cancers_;_ numbers of people with most site-specific cancers were small. Similar associations were observed when omitting BMI as a covariate (Additional file 1: Figure S4), but associations were generally stronger in those with pre-baseline disease than the main cohort (Additional file 1: Figure S5; characteristics presented in Table S6). Comparing mortality associations of the three summary scores, hazard ratios for mortality per 1 SD increment were consistently strongest for PAEE_SR_ (Fig. 3). The IPAQ and LTPA+OPA scores showed no association with cancer mortality in contrast to PAEE_SR_. Additionally adjusting for sleep in the Cox model did not meaningfully alter associations for IPAQ and LTPA+OPA scores (data not shown).

There were dose-response associations across quartiles of PAEE_SR_, with lower hazard in higher quartiles, and attenuation of the effect with additional adjustment for BMI (Supplementary Table S5). There was a non-linear inverse association of PAEE_SR_ with all-cause mortality (Supplementary Fig. S3), with steeper gradient of the relationship moving from the least active individual to ~ 15 kJ/day/kg PAEE_SR_, and shallower gradient above that level with greater uncertainty.

## Discussion

This study reports the reliability and validity of PAEE predicted from a range of self-reported behaviours using a network harmonisation approach which included calibration to 7-day wrist accelerometry in approximately 100,000 free-living individuals. Our findings suggest that this method of combining behavioural data in UK Biobank produces PAEE values suitable for ranking individuals (based on Spearman’s rank-order correlation) and demonstrates predictive validity when examining associations with morbidity and mortality, for example showing 14% lower mortality for individuals accumulating PAEE equivalent to meeting the lower World Health Organization physical activity guidelines [35]. However there are challenges with interpretation on an absolute scale due to marked under- and over-estimation at the exposure extremes.

Test-retest reliability of PAEE_SR_ outperformed MET-minute scores from IPAQ and LTPA+OPA and many previous self-reported estimates [2] despite an average of 4 years between baseline and repeat assessment, during which it might be expected for physical activity to decline in this population. We were not able to examine whether there were ‘true’ within-individual changes in PAEE between time-points using a criterion, but accounting for such changes would likely serve to improve reliability coefficients observed here. It is encouraging to note that although the behaviours demonstrated relatively poor test-retest reliability in isolation, combining them provides an estimate of PAEE_SR_ which seems to better reflect a habitual level of activity.

In the separate DLW validation study, PAEE_SR_ showed a non-significant 2% underestimation and explained 27% variance in PAEE_DLW_. This compares favourably to the relative validity of scores from IPAQ and LTPA+OPA reported here, as well as self-reported activity volume in previous work [2], with stronger criterion validity than estimates from IPAQ [6, 37, 38] and RPAQ [7, 8], on which the questions are based. This may be explained by inclusion of a more comprehensive and complimentary list of physical activity behaviours, as well as sleep and sedentary behaviours which also provide information about the total volume of movement each day. Our validation study findings indicate that PAEE_SR_ explains much higher levels of variance in the ‘true’ volume of physical activity assessed by PAEE_DLW_, and this is reflected in stronger associations with mortality in UK Biobank compared with IPAQ and LTPA+OPA, which were more attenuated.

Estimation errors were strongly negatively correlated with the criterion PAEE_DLW_, i.e. displaying regression to the mean which is a consequence of using a relatively weak self-report instrument and prediction equations explaining relatively low levels of variance in wrist ENMO. The explanatory power of our models could have been strengthened using additional predictors (e.g. age, adiposity, etc.), but these are not directly representative of activity, and inclusion of more complicated predictors could hinder the transferability of newly derived models even if the relevant behavioural variables are available. Therefore, in order to make results more useful in answering epidemiological questions about the role of physical activity, we employed a model using behavioural data. Weak prediction models with a large constant narrowed the observed range of predicted values substantially resulting in overestimation at the lower end and underestimation for more active individuals, widening the 95% limits of agreement. The component of PAEE_SR_ from the constant is mathematically insensitive to differences in behaviour between individuals and does not influence correlations with criterion PAEE_DLW_ or health associations; it does, however, impact interpretation of the exposure on an absolute scale, which presents a challenge for translation of observed associations with mortality to public health recommendations [39]. To facilitate such interpretation, we marginalised PAEE_SR_ by subtracting the level of exposure of the least active individual from all participants in the analytical sample. The resulting dose-response curve for all-cause mortality is consistent with messages emphasising greater benefits of increasing PAEE at the lower end of the exposure range [40]. Future work should explore methods to remedy these prediction errors and make use of alterative statistical approaches which combine data to give an integrated score [41]; the present study aimed to predict physical activity volume rather than characterise the overall pattern of health-related behaviours.

Limitations of this study include a healthy volunteer selection bias in UK Biobank such that it is not representative of the general population [42]; the accelerometer sub-cohort may also suffer from selection bias, although no major differences in self-reported behaviours or PAEE_SR_ were observed here. There was an average 5.7 year gap between baseline self-reported behaviours and the accelerometer data used for calibration. We cannot rule out that physical activity may have changed in this time, although PAEE_SR_ in the repeat assessment sub-cohort was relatively stable over a similar period and we accounted for change in age and season between these time points when deriving the prediction equations. The generalisability of prediction equations to those who did not survive until the accelerometry sub-cohort commenced must also be considered. This would be a concern if individuals who died during this period exhibited different relationships between self-reported behaviours and wrist ENMO, rather than just different behaviours. Given the size of the calibration samples, we argue that the heterogeneity of relationships included when deriving the models is sufficient. Furthermore, the accelerometry sub-study occurred over a number of years, meaning that some individuals who died relatively early in the follow-up period would have been included. Further work is necessary to explore the effects of using calibration equations with relatively weak self-report instruments, as these will be important for future harmonisation efforts (e.g. for synthesis of data from studies using different self-report methods). In particular, it is necessary to understand how calibrated and non-calibrated self-reported data should be used to estimate associations with disease outcomes across the full dose range, given the challenges of interpretation we have reported. Strengths of the work include use of PAEE_DLW_ for examining validity, and propagation of the uncertainty (prediction errors) accrued at each step of our method for estimating PAEE to the analyses of associations with disease outcomes. Wrist accelerometry has strong validity compared to PAEE_DLW_ [4], but is not available in the whole UK Biobank cohort and there is much less follow-up time in the sub-cohort where the measure is available. We used a robust criterion to calibrate and harmonise 14 self-report variables, with the added advantage that the necessary self-report data exist for approximately 475,000 participants, permitting use as an exposure, outcome, or covariate in future analyses.

## Conclusions

In conclusion, we have successfully utilised a network harmonisation approach to exploit the diverse behavioural data in UK Biobank and derive an overall summary estimate of PAEE. The PAEE_SR_ variable has good reliability and validity for ranking individuals compared with other self-report methods. It is the only estimate of PAEE available in the main UK Biobank cohort which has been tested against the gold-standard DLW-based criterion, showing no mean bias but a systematic bias at individual level stemming from inherent weaknesses of the self-report data. It does however have predictive validity in that it is prospectively associated with morbidity and mortality, and in a way that can be interpreted in a public health framework.

## Supplementary information

**Additional file 1: Table S1.** Questions used to generate domain-specific and composite behavioural variables. **Table S2.** Calculation of comparison summary scores using METs. **Table S3.** International Classification of Diseases 10th edition (ICD-10) codes for outcome definition. **Table S4.** Mutually adjusted sex-specific coefficients (standard errors) for prediction of average daily wrist acceleration (m-g) from 14 self-reported behaviours. **Table S5.** Hazard ratio and 95% confidence interval for fatal and non-fatal outcomes by quartile of PAEE_SR_ in UK Biobank. **Table S6** Baseline characteristics of participants with prevalent chronic disease in UK Biobank. **Figure S1.** Exclusions and sample sizes for analyses. **Figure S2.** Differences between physical activity energy expenditure predicted from self-report (PAEE_SR_) and doubly labelled water based PAEE (PAEE_DLW_), plotted against their mean. **Figure S3.** Hazard ratio and 95% confidence intervals for association between physical activity energy expenditure predicted from self-report (PAEE_SR_) and disease outcomes in UK Biobank. **Figure S4.** Hazard ratio (HR) and 95% confidence interval (CI) for linear associations of physical activity energy expenditure predicted from self-report (PAEE_SR_, per 5 kJ/day/kg increments) with fatal and non-fatal outcomes in UK Biobank. **Figure S5.** Hazard ratio (HR) and 95% confidence interval (CI) for linear associations of physical activity energy expenditure predicted from self-report (PAEE_SR_, per 5 kJ/day/kg increments) with fatal and non-fatal outcomes in UK Biobank.

## Data Availability

The UK Biobank data (Application Number 20684) that support the findings of this study are available to all bona fide researchers for health related research that is in the public interest, https://www.ukbiobank.ac.uk/register-apply/. The Biobank Validation Study data that support the findings of this study are available on request at https://epi-meta.mrc-epid.cam.ac.uk/.

## Abbreviations

BMI: Body mass index
CI: Confidence interval
COPD: Chronic obstructive pulmonary disease
CVD: Cardiovascular disease
DIY: Do-it-yourself
DLW: Doubly labelled water
ENMO: Euclidean norm minus one
HR: Hazard ratio
ICD-10: International Classification of Diseases 10th edition
IHD: Ischaemic heart disease
IPAQ: International Physical Activity Questionnaire
LTPA+OPA: Leisure-time and occupational physical activity
MET: Metabolic equivalent of task
MVPA: Moderate-to-vigorous physical activity
PAEE: Physical activity energy expenditure
PAEE_DLW_: Physical activity energy expenditure from doubly labelled water
PAEE_SR_: Physical activity energy expenditure predicted from self-report
RMSE: Root mean square error
RPAQ: Recent Physical Activity Questionnaire
SD: Standard deviation
SE: Standard error
TV: Television
