# Supplementary material for: Estimating physical activity from self-reported behaviours in large-scale population studies using network harmonisation: findings from UK Biobank and associations with disease outcomes
Source: Int J Behav Nutr Phys Act. 2020 Mar 16;17:40. doi: 10.1186/s12966-020-00937-4 (PMC7074990; doi:10.1186/s12966-020-00937-4)
Supplement: Supplementary file 1 — Additional file 1: Table S1. Questions used to generate domain-specific and composite behavioural variables. Table S2. Calculation of comparison summary scores using METs. Table S3. International Classification of Diseases 10th edition (ICD-10) codes for outcome definition. Table S4. Mutually adjusted sex-specific coefficients (standard errors) for prediction of average daily wrist acceleration (m-g) from 14 self-reported behaviours. Table S5. Hazard ratio and 95% confidence interval for fatal and non-fatal outcomes by quartile of PAEESR in UK Biobank. Table S6 Baseline characteristics of participants with prevalent chronic disease in UK Biobank. Figure S1. Exclusions and sample sizes for analyses. Figure S2. Differences between physical activity energy expenditure predicted from self-report (PAEESR) and doubly labelled water based PAEE (PAEEDLW), plotted against their mean. Figure S3. Hazard ratio and 95% confidence intervals for association between physical activity energy expenditure predicted from self-report (PAEESR) and disease outcomes in UK Biobank. Figure S4. Hazard ratio (HR) and 95% confidence interval (CI) for linear associations of physical activity energy expenditure predicted from self-report (PAEESR, per 5 kJ/day/kg increments) with fatal and non-fatal outcomes in UK Biobank. Figure S5. Hazard ratio (HR) and 95% confidence interval (CI) for linear associations of physical activity energy expenditure predicted from self-report (PAEESR, per 5 kJ/day/kg increments) with fatal and non-fatal outcomes in UK Biobank. [file 12966_2020_937_MOESM1_ESM.docx]

**Table S1** Questions used to generate domain-specific and composite behavioural variables.

| Variable | Units | Domain | Original question(s) | Field ID |
| --- | --- | --- | --- | --- |
| Heavy physical work | Min/day | Occupation-time | - Does your work involve heavy manual or physical work? - In a typical WEEK, how many hours do you spend at work? (Do not include hours travelling to and from work) | 816  767 |
| Walking/standing work | Min/day | Occupation-time | - Does your work involve walking or standing for most of the time? - In a typical WEEK, how many hours do you spend at work? (Do not include hours travelling to and from work) | 806  767 |
| Sedentary work | Min/day | Occupation-time | Difference between total work time and sum of physical and standing/walking work time   - In a typical WEEK, how many hours do you spend at work? (Do not include hours travelling to and from work) | 767 |
| MVPA | Min/day | Total activity of at least moderate intensity | - In a typical WEEK, on how many days did you walk for at least 10 minutes at a time? (Include walking that you do at work, travelling to and from work, and for sport or leisure) | 864 |
|  |  |  | - How many minutes did you usually spend walking on a typical DAY? | 874 |
|  |  |  | - In a typical WEEK, on how many days did you do 10 minutes or more of moderate physical activities like carrying light loads, cycling at normal pace? (Do not include walking) | 884 |
|  |  |  | - How many minutes did you usually spend doing moderate activities on a typical DAY? | 894 |
|  |  |  | - In a typical WEEK, how many days did you do 10 minutes or more of vigorous physical activity? (These are activities that make you sweat or breathe hard such as fast cycling, aerobics, heavy lifting) | 904 |
|  |  |  | - How many minutes did you usually spend doing vigorous activities on a typical DAY? | 914 |
| Walking for pleasure | Min/day | Leisure-time | - How many times in the last 4 weeks did you go walking for pleasure? | 971 |
|  |  |  | - Each time you went walking for pleasure, about how long did you spend doing it? | 981 |
| Strenuous sports | Min/day | Leisure-time | - How many times in the last 4 weeks did you do strenuous sports? | 991 |
|  |  |  | - Each time you did strenuous sports, about how long did you spend doing it? | 1001 |
| Other exercises | Min/day | Leisure-time | - How many times in the last 4 weeks did you do other exercises such as swimming, cycling, keep fit? | 3637 |
|  |  |  | - Each time you did other exercises such as swimming, cycling, keep fit, about how long did you spend doing them? | 3647 |
| Light DIY | Min/day | Leisure-time | - How many times in the last 4 weeks did you do light DIY? | 1011 |
|  |  |  | - Each time you did light DIY, about how long did you spend doing it? | 1021 |
| Heavy DIY | Min/day | Leisure-time | - How many times in the last 4 weeks did you do heavy DIY? | 2624 |
|  |  |  | - Each time you did heavy DIY, about how long did you spend doing it? | 2634 |
| TV viewing | Hours/day | Screen-time | - In a typical DAY, how many hours do you spend watching TV? | 1070 |
| Computer use | Hours/day | Screen-time | - In a typical DAY, how many hours do you spend using the computer? (Do not include using a computer at work; put 0 if you do not spend any time doing it) | 1080 |
| Getting about | Categorical | Transport | - In the last 4 weeks, which forms of transport have you used most often to get about? (Not including any journeys to and from work; you can select more than one answer) Car/motor vehicle, walk, public transport, cycle. | 6162 |
| Commuting | Categorical | Transport | - What types of transport do you use to get to and from work? (You can select more than one answer) Car/motor vehicle, walk, public transport, cycle | 6143 |
| Sleep | Hours/day | Sleep | - About how many hours sleep do you get in every 24 hours? (please include naps) | 1160 |

DIY=do-it-yourself; MVPA=moderate-to-vigorous physical activity; TV=television. Field ID relates to UK Biobank Showcase <http://biobank.ctsu.ox.ac.uk/crystal/>

**Table S2** Calculation of comparison summary scores using METs.

| IPAQ score in MET-minutes/day is the sum of: | Walking (minutes/day) * 3.3 METs  Moderate activity (minutes/day) * 4.0 METs  Vigorous activity (minutes/day) * 8.0 METs |
| --- | --- |
| LTPA+OPA score in MET-minutes/day is the sum of: | Walking for pleasure (minutes/day) * 3.3 METs  Strenuous sports (minutes/day) * 8.0 METs  Other exercises (minutes/day) * 4.5 METs  Light DIY (minutes/day) * 2.25 METs  Heavy DIY (minutes/day) * 4.5 METs  Heavy physical work (minutes/day) * 4.5 METs  Walking/standing work (minutes/day) * 2.25 METs |

DIY=do-it-yourself; IPAQ=International Physical Activity Questionnaire; LTPA+OPA=leisure-time and occupational physical activity; MET=metabolic equivalent of task.

**Table S3** International Classification of Diseases 10th edition (ICD-10) codes for outcome definition.

| Outcome | ICD-10 code(s) |
| --- | --- |
| Heart failure | I50, I110, I130, I132 |
| Stroke | I60-166 |
| Ischaemic heart disease | I20-I25 |
| Atrial fibrillation | I48 |
| All cardiovascular disease | I5-I9, I10-I89 |
| Chronic obstructive pulmonary disease | J44 |
| All respiratory disease | J00-J99 |
| Breast cancer | C50 |
| Prostate cancer | C61 |
| Colon cancer | C18 |
| Malignant melanoma cancer | C43 |
| Bladder cancer | C67 |
| Endometrial cancer | C54 |
| Rectum cancer | C20 |
| Lung cancer | C34 |
| Kidney cancer | C64-C65 |
| Myeloma cancer | C90 |
| Oesophageal cancer | C15 |
| Myeloid leukaemia cancer | C92 |
| Liver cancer | C22-C24 |
| Gastric cardia cancer | C160 |
| Head or neck cancer | C760 |
| All cancer | C, D00-D49 |
| Cardiovascular mortality | I5-I9, I10-I89 |
| Respiratory disease mortality | J00-J99 |
| Cancer mortality | C, D00-D49 |

**Table S4** Mutually adjusted sex-specific coefficients (standard errors) for prediction of average daily wrist acceleration (m-g) from 14 self-reported behaviours.

| Variable | Women (n=52,507) | Men (n=40,918) |
| --- | --- | --- |
| Minutes per day of: |  |  |
| Heavy physical work (log*_e_*) | 0.327 (0.029) | 0.512 (0.030) |
| Walking/standing work (log*_e_*) | 0.261 (0.018) | 0.213 (0.022) |
| Sedentary work (log*_e_*) | 0.106 (0.013) | 0.141 (0.017) |
| MVPA (log*_e_*) | 0.494 (0.028) | 0.617 (0.037) |
| Walking for pleasure (log*_e_*) | 0.473 (0.027) | 0.298 (0.031) |
| Strenuous sports (log*_e_*) | 1.18 (0.050) | 1.23 (0.046) |
| Other exercises (log*_e_*) | 0.563 (0.026) | 0.651 (0.029) |
| Light DIY (log*_e_*) | -0.019 (0.025) | 0.069 (0.028) |
| Heavy DIY (log*_e_*) | 0.383 (0.033) | 0.336 (0.031) |
| Hours per day of: |  |  |
| Television viewing (log*_e_*) | -2.29 (0.077) | -2.22 (0.092) |
| Computer use (log*_e_*) | -1.47 (0.074) | -1.59 (0.084) |
| Getting about method: |  |  |
| Car or public transportation | Reference | Reference |
| Mixed use | 0.102 (0.071) | 0.180 (0.084) |
| Walking or cycling | 0.887 (0.134) | 1.34 (0.167) |
| Commuting method: |  |  |
| Car or public transportation | Reference | Reference |
| Mixed use | 0.963 (0.123) | 1.00 (0.146) |
| Walking or cycling | 1.47 (0.174) | 1.46 (0.225) |
| Hours per day of sleep: |  |  |
| ≤5.0 | -0.636 (0.177) | -0.002 (0.257) |
| 6.0 | -0.351 (0.095) | 0.177 (0.110) |
| 7.0 | Reference | Reference |
| 8.0 | -0.601 (0.077) | -0.806 (0.095) |
| ≥9.0 | -1.93 (0.130) | -2.06 (0.168) |
| Constant | 28.9 (0.253) | 27.2 (0.309) |

DIY=do-it-yourself; MVPA=moderate-to-vigorous physical activity; TV=television.

Natural log transformations of self-reported behaviours used log*_e_* (x+1).

**Table S5** Hazard ratio and 95% confidence interval for fatal and non-fatal outcomes by quartile of PAEE_SR_ in UK Biobank.

|  | Quartile 1 | Quartile 2 | Quartile 3 | Quartile 4 |
| --- | --- | --- | --- | --- |
| PAEE_SR_ (kJ/day/kg) |  |  |  |  |
| Range | 0.0-11.9 | 11.9-14.3 | 14.3-16.9 | 16.9-35.9 |
| Median | 10.3 | 13.2 | 15.5 | 18.9 |
| ***All-cause mortality*** | | | | |
| Participants/events | 93,588/3,802 | 93,588/2,379 | 93,588/1,858 | 93,588/1,333 |
| Person-years | 821,881 | 826,893 | 829,847 | 833,151 |
| HR (95%CI)^A^ | 1 | 0.84 (0.80 to 0.88) | 0.82 (0.77 to 0.87) | 0.76 (0.71 to 0.82) |
| HR (95%CI)^B^ | 1 | 0.85 (0.81 to 0.90) | 0.84 (0.79 to 0.89) | 0.79 (0.73 to 0.85) |
| ***Cancer mortality*** | | | | |
| Participants/events | 93,588/2,131 | 93,588/1,475 | 93,588/1,208 | 93,588/855 |
| Person-years | 821,881 | 826,893 | 829,847 | 833,151 |
| HR (95%CI)^A^ | 1 | 0.88 (.82 to .94) | 0.88 (0.82 to 0.95) | 0.81 (0.74 to 0.88) |
| HR (95%CI)^B^ | 1 | 0.89 (.84 to .96) | 0.90 (0.84 to 0.97) | 0.83 (0.76 to 0.91) |
| ***CVD mortality*** | | | | |
| Participants/events | 93,588/1,230 | 93,588/689 | 93,588/511 | 93,588/344 |
| Person-years | 821,881 | 826,893 | 829,847 | 833,151 |
| HR (95%CI)^A^ | 1 | 0.85 (0.78 to 0.94) | .84 (0.75 to 0.94) | 0.77 (0.67 to 0.88) |
| HR (95%CI)^B^ | 1 | 0.90 (0.82 to 0.99) | .91 (0.82 to 1.02) | 0.85 (0.75 to 0.98) |
| ***Respiratory disease mortality*** | | | | |
| Participants/events | 93,588/980 | 93,588/520 | 93,588/356 | 93,588/212 |
| Person-years | 821,881 | 826,893 | 829,847 | 833,151 |
| HR (95%CI)^A^ | 1 | 0.79 (0.71 to 0.88) | 0.73 (0.64 to 0.83) | 0.61 (0.52 to 0.72) |
| HR (95%CI)^B^ | 1 | 0.80 (0.71 to 0.89) | 0.74 (0.65 to 0.84) | 0.62 (0.53 to 0.73) |
| ***All CVD incidence*** | | | | |
| Participants/events | 84,539/14,576 | 87,398/11,474 | 88,531/9,692 | 89,800/8,046 |
| Person-years | 669,455 | 715,333 | 736,720 | 759,434 |
| HR (95%CI)^A^ | 1 | 0.91 (0.89 to 0.93) | 0.87 (0.85 to 0.89) | 0.84 (0.81 to 0.86) |
| HR (95%CI)^B^ | 1 | 0.95 (0.92 to 0.97) | 0.93 (0.90 to 0.96) | 0.92 (0.89 to 0.95) |
| ***IHD incidence*** | | | | |
| Participants/events | 92,536/2,510 | 92,929/1,727 | 93,035/1,406 | 93,199/1,226 |
| Person-years | 799,883 | 812,126 | 817,643 | 823,207 |
| HR (95%CI)^A^ | 1 | 0.90 (0.84 to 0.96) | .86 (0.80 to 0.92) | .87 (0.80 to 0.94) |
| HR (95%CI)^B^ | 1 | 0.93 (0.88 to 0.99) | .91 (0.85 to 0.98) | .94 (0.87 to 1.02) |
| ***Atrial fibrillation incidence*** | | | | |
| Participants/events | 92,727/1,786 | 92,982/1,312 | 93,124/997 | 93,233/799 |
| Person-years | 805,215 | 814,759 | 820,330 | 825,478 |
| HR (95%CI)^A^ | 1 | 0.94 (0.88 to 1.02) | .87 (0.80 to 0.94) | .86 (0.79 to 0.95) |
| HR (95%CI)^B^ | 1 | 1.01 (0.94 to 1.08) | .96 (0.89 to 1.05) | .99 (0.90 to 1.09) |
| ***Stroke incidence*** | | | | |
| Participants/events | 93,371/604 | 93,456/433 | 93,469/382 | 93,508/278 |
| Person-years | 816,730 | 823,184 | 826,559 | 830,575 |
| HR (95%CI)^A^ | 1 | 0.92 (0.81 to 1.04) | .97 (0.84 to 1.11) | .86 (0.73 to 1.00) |
| HR (95%CI)^B^ | 1 | 0.93 (0.82 to 1.05) | .98 (0.85 to 1.12) | .87 (0.74 to 1.03) |
| ***Heart failure incidence*** | | | | |
| Participants/events | 93,416/542 | 93,499/349 | 93,517/245 | 93,547/162 |
| Person-years | 817,407 | 823,924 | 827,563 | 831,489 |
| HR (95%CI)^A^ | 1 | 0.92 (0.80 to 1.05) | 0.80 (0.69 to 0.94) | 0.67 (0.55 to 0.81) |
| HR (95%CI)^B^ | 1 | 0.99 (0.86 to 1.13) | 0.90 (0.77 to 1.06) | 0.78 (0.64 to 0.96) |
| ***All respiratory disease incidence*** | | | | |
| Participants/events | 90,327/6,493 | 91,292 /5,001 | 91,631/4,388 | 91,961/3,872 |
| Person-years | 762,662 | 783,222 | 791,827 | 800,085 |
| HR (95%CI)^A^ | 1 | 0.86 (0.83 to 0.89) | 0.82 (0.79 to 0.85) | 0.79 (0.76 to 0.83) |
| HR (95%CI)^B^ | 1 | 0.89 (0.86 to 0.92) | 0.86 (0.83 to 0.90) | 0.86 (0.82 to 0.90) |
| ***COPD incidence**** | | | | |
| Participants/events | 93,103/1,245 | 93,398/642 | 93,472/434 | 93,525/253 |
| Person-years | 811,511 | 821,795 | 826,297 | 830,892 |
| HR (95%CI)^A^ | 1 | 0.78 (0.71 to 0.86) | 0.68 (0.61 to 0.76) | 0.55 (0.47 to 0.64) |
| HR (95%CI)^B^ | 1 | 0.78 (0.71 to 0.86) | 0.69 (0.61 to 0.78) | 0.56 (0.48 to 0.65) |
| ***All cancer incidence*** | | | | |
| Participants/events | 90,372/6,578 | 90,812/5,935 | 91,061/5,699 | 91,400/4,822 |
| Person-years | 760,835 | 773,061 | 779,074 | 789,498 |
| HR (95%CI)^A^ | 1 | 0.96 (0.92 to 0.99) | 0.99 (0.95 to 1.02) | 0.92 (0.88 to 0.95) |
| HR (95%CI)^B^ | 1 | 0.97 (0.93 to 1.00) | 1.00 (0.96 to 1.04) | 0.94 (0.90 to 0.98) |
| ***Breast cancer incidence*** | | | | |
| Participants/events | 44,046/609 | 54,713/682 | 56,901/740 | 51,200/553 |
| Person-years | 385,332 | 480,459 | 500,852 | 452,658 |
| HR (95%CI)^A^ | 1 | 0.90 (0.81 to 1.01) | 0.95 (0.85 to 1.07) | 0.81 (0.71 to 0.92) |
| HR (95%CI)^B^ | 1 | 0.92 (0.82 to 1.02) | 0.97 (0.87 to 1.09) | 0.83 (0.73 to 0.95) |
| ***Prostate cancer incidence*** | | | | |
| Participants/events | 48,993/667 | 38,406/493 | 36,257/422 | 42,052/367 |
| Person-years | 424,962 | 336,012 | 318,958 | 372,426 |
| HR (95%CI)^A^ | 1 | 1.06 (0.95 to 1.19) | 1.13 (0.99 to 1.28) | 1.08 (0.94 to 1.24) |
| HR (95%CI)^B^ | 1 | 1.05 (0.93 to 1.18) | 1.11 (0.97 to 1.26) | 1.05 (0.91 to 1.21) |
| ***Colon cancer incidence*** | | | | |
| Participants/events | 93,453/237 | 93,504/204 | 93,490/156 | 93,536/122 |
| Person-years | 819,043 | 824,641 | 827,696 | 831,469 |
| HR (95%CI)^A^ | 1 | 0.99 (0.82 to 1.19) | 0.87 (0.70 to 1.07) | 0.80 (0.63 to 1.02) |
| HR (95%CI)^B^ | 1 | 1.00 (0.83 to 1.21) | 0.89 (0.72 to 1.10) | 0.83 (0.65 to 1.07) |
| ***Malignant melanoma cancer incidence*** | | | | |
| Participants/events | 93,528/145 | 93,520/151 | 93,539/135 | 93,534/129 |
| Person-years | 820,158 | 825,020 | 828,194 | 831,455 |
| HR (95%CI)^A^ | 1 | 1.08 (0.86 to 1.36) | 1.00 (0.79 to 1.28) | 1.02 (0.78 to 1.32) |
| HR (95%CI)^B^ | 1 | 1.08 (0.86 to 1.36) | 1.00 (0.78 to 1.28) | 1.01 (0.78 to 1.32) |
| ***Bladder cancer incidence*** | | | | |
| Participants/events | 93,504/189 | 93,526/133 | 93,552/116 | 93,550/76 |
| Person-years | 819,686 | 825,207 | 828,404 | 831,873 |
| HR (95%CI)^A^ | 1 | 0.99 (0.79 to 1.24) | 1.07 (0.83 to 1.36) | 0.86 (0.64 to 1.16) |
| HR (95%CI)^B^ | 1 | 1.00 (0.80 to 1.25) | 1.08 (0.84 to 1.38) | 0.88 (0.65 to 1.18) |
| ***Endometrial cancer incidence*** | | | | |
| Participants/events | 93,549/86 | 93,557/94 | 93,546/92 | 93,560/67 |
| Person-years | 820,563 | 825,585 | 828,439 | 832,012 |
| HR (95%CI)^A^ | 1 | 0.93 (0.70 to 1.25) | 0.92 (0.68 to 1.26) | 0.82 (0.57 to 1.17) |
| HR (95%CI)^B^ | 1 | 1.13 (0.84 to 1.52) | 1.24 (0.90 to 1.70) | 1.22 (0.85 to 1.78) |
| ***Rectum cancer incidence*** | | | | |
| Participants/events | 93531/96 | 93539/92 | 93541/69 | 93555/60 |
| Person-years | 820,400 | 825,512 | 828,522 | 831,981 |
| HR (95%CI)^A^ | 1 | 1.13 (0.85 to 1.51) | 0.96 (0.69 to 1.33) | 0.96 (0.67 to 1.38) |
| HR (95%CI)^B^ | 1 | 1.13 (0.85 to 1.51) | 0.96 (0.69 to 1.33) | 0.96 (0.67 to 1.39) |
| ***Lung cancer incidence*** | | | | |
| Participants/events | 93,530/81 | 93,541/71 | 93,548/53 | 93,561/37 |
| Person-years | 820,486 | 825,695 | 828,708 | 832,223 |
| HR (95%CI)^A^ | 1 | 1.11 (0.80 to 1.53) | 0.97 (0.67 to 1.40) | 0.90 (0.58 to 1.39) |
| HR (95%CI)^B^ | 1 | 1.09 (0.79 to 1.51) | 0.95 (0.65 to 1.37) | 0.87 (0.56 to 1.36) |
| ***Kidney cancer incidence*** | | | | |
| Participants/events | 93,548/82 | 93,554/78 | 93,568/48 | 93,581/33 |
| Person-years | 820,640 | 825,688 | 828,869 | 832,331 |
| HR (95%CI)^A^ | 1 | 1.08 (0.79 to 1.48) | 0.73 (0.50 to 1.06) | 0.57 (0.37 to 0.89) |
| HR (95%CI)^B^ | 1 | 1.14 (0.83 to 1.56) | 0.79 (0.54 to 1.15) | 0.64 (0.41 to 1.00) |
| ***Myeloma cancer incidence*** | | | | |
| Participants/events | 93,570/41 | 93,576/41 | 93,570/35 | 93,577/29 |
| Person-years | 820,998 | 826,012 | 828,972 | 832,342 |
| HR (95%CI)^A^ | 1 | 1.17 (0.76 to 1.81) | 1.09 (0.67 to 1.75) | 1.04 (0.61 to 1.76) |
| HR (95%CI)^B^ | 1 | 1.19 (0.77 to 1.85) | 1.12 (0.69 to 1.81) | 1.08 (0.63 to 1.85) |
| ***Oesophageal cancer incidence*** | | | | |
| Participants/events | 93,552/39 | 93,570/25 | 93,573/21 | 93,576/24 |
| Person-years | 820,923 | 826,089 | 829,069 | 832,353 |
| HR (95%CI)^A^ | 1 | 0.87 (0.53 to 1.45) | 0.95 (0.54 to 1.65) | 1.42 (0.80 to 2.54) |
| HR (95%CI)^B^ | 1 | 0.95 (0.57 to 1.57) | 1.07 (0.61 to 1.87) | 1.67 (0.93 to 3.01) |
| ***Myeloid leukaemia cancer incidence*** | | | | |
| Participants/events | 93,584/16 | 93,583/14 | 93,581/16 | 93,582/12 |
| Person-years | 821,265 | 826,237 | 829,140 | 832,465 |
| HR (95%CI)^A^ | 1 | 0.88 (0.43 to 1.82) | 0.96 (0.46 to 2.02) | 0.81 (0.35 to 1.85) |
| HR (95%CI)^B^ | 1 | 0.91 (0.44 to 1.89) | 1.02 (0.48 to 2.14) | 0.87 (0.38 to 2.02) |
| ***Liver cancer incidence*** | | | | |
| Participants/events | 93,578/20 | 93,571/5 | 93,581/11 | 93,583/13 |
| Person-years | 821,186 | 826,196 | 829,170 | 832,476 |
| HR (95%CI)^A^ | 1 | 0.32 (0.12 to 0.87) | 0.77 (0.35 to 1.72) | 1.20 (0.53 to 2.69) |
| HR (95%CI)^B^ | 1 | 0.33 (0.12 to 0.90) | 0.81 (0.36 to 1.83) | 1.29 (0.57 to 2.94) |
| ***Gastric cardia cancer incidence*** | | | | |
| Participants/events | 93,576/12 | 93,577/9 | 93,586/10 | 93,584/9 |
| Person-years | 821,240 | 826,221 | 829,197 | 832,483 |
| HR (95%CI)^A^ | 1 | 1.16 (0.49 to 2.74) | 1.74 (0.72 to 4.17) | 2.06 (0.78 to 5.45) |
| HR (95%CI)^B^ | 1 | 1.24 (0.52 to 2.92) | 1.91 (0.79 to 4.65) | 2.34 (0.87 to 6.29) |

CI=confidence interval; COPD=chronic obstructive pulmonary disease; CVD=cardiovascular disease; HR=hazard ratio; IHD=ischaemic heart disease; PAEE_SR_=physical activity energy expenditure predicted from self-report, rescaled to the level of physical activity above the least active participant.

^A^Model 1: age (as timescale), sex, ethnicity (baseline hazard stratification), Townsend deprivation index, highest educational level, employment status, alcohol drinking status (baseline hazard stratification), smoking status, salt added to food, oily fish intake, fruit and vegetable intake, processed and red meat intake, parental history of cancer, parental history of [heart disease, stroke, hypertension or diabetes], use of blood pressure medication, use of cholesterol lowering medication, doctor-diagnosed diabetes or treatment with insulin.

^B^Model 2: Model 1 plus body mass index.

*COPD incidence likely only represents the most severe cases as only approximately 25% of COPD cases are picked up in Hospital Episode Statistics data, compared to national surveys (1).

**Table S6** Baseline characteristics of participants with prevalent chronic disease in UK Biobank.

|  | Participants with pre-baseline disease |
| --- | --- |
| Analysis sample (n) | 77,843 |
| Age at baseline (years) | 60 (7) |
| Proportion of women | 51% |
| Weight (kg) | 80 (16) |
| Body mass index (kg/m^2^) | 28 (5) |
| Minutes per day of: |  |
| Heavy physical work | 15 (44) |
| Walking/standing work | 39 (81) |
| Sedentary work | 68 (121) |
| MVPA | 86 (107) |
| Walking for pleasure | 15 (23) |
| Strenuous sports | 2 (8) |
| Other exercises | 8 (17) |
| Light DIY | 10 (25) |
| Heavy DIY | 6 (19) |
| Hours per day of: |  |
| Television viewing | 3 (2) |
| Computer use | 1 (1) |
| Getting about method: |  |
| Car or public transportation | 51% |
| Mixed use | 41% |
| Walking or cycling | 8% |
| Commuting method: |  |
| Car or public transportation | 92% |
| Mixed use | 5% |
| Walking or cycling | 3% |
| Hours per day of sleep: |  |
| ≤5.0 | 7% |
| 6.0 | 19% |
| 7.0 | 35% |
| 8.0 | 29% |
| ≥9.0 | 10% |
| PAEE_SR_ (kJ/day/kg) | 46 (4) |
| IPAQ scoring (MET-minutes/day) | 354 (447) |
| LTPA+OPA scoring (MET-minutes/day) | 302 (383) |

Pre-baseline disease refers to pre-baseline hospital episodes of stroke, ischaemic heart disease, respiratory disease, or cancer; self-report of doctor diagnosis of stroke, ischaemic heart disease, or cancer.

DIY=do-it-yourself; DLW=doubly labelled water; IPAQ=International Physical Activity Questionnaire; LTPA+OPA=leisure-time and occupational physical activity; MET=metabolic equivalent of task; MET=metabolic equivalent of task; MVPA=moderate-to-vigorous physical activity; PAEE_SR_=physical activity energy expenditure predicted from self-report.

Values are means (standard deviations) unless otherwise stated.

|  |
| --- |
| **Fig S1.** Exclusions and sample sizes for analyses.  DLW=doubly labelled water; ENMO=Euclidean norm minus one (m-g).  *Stroke, ischaemic heart disease, respiratory disease, or cancer. |

**Stroke, ischaemic heart disease, or cancer.

| **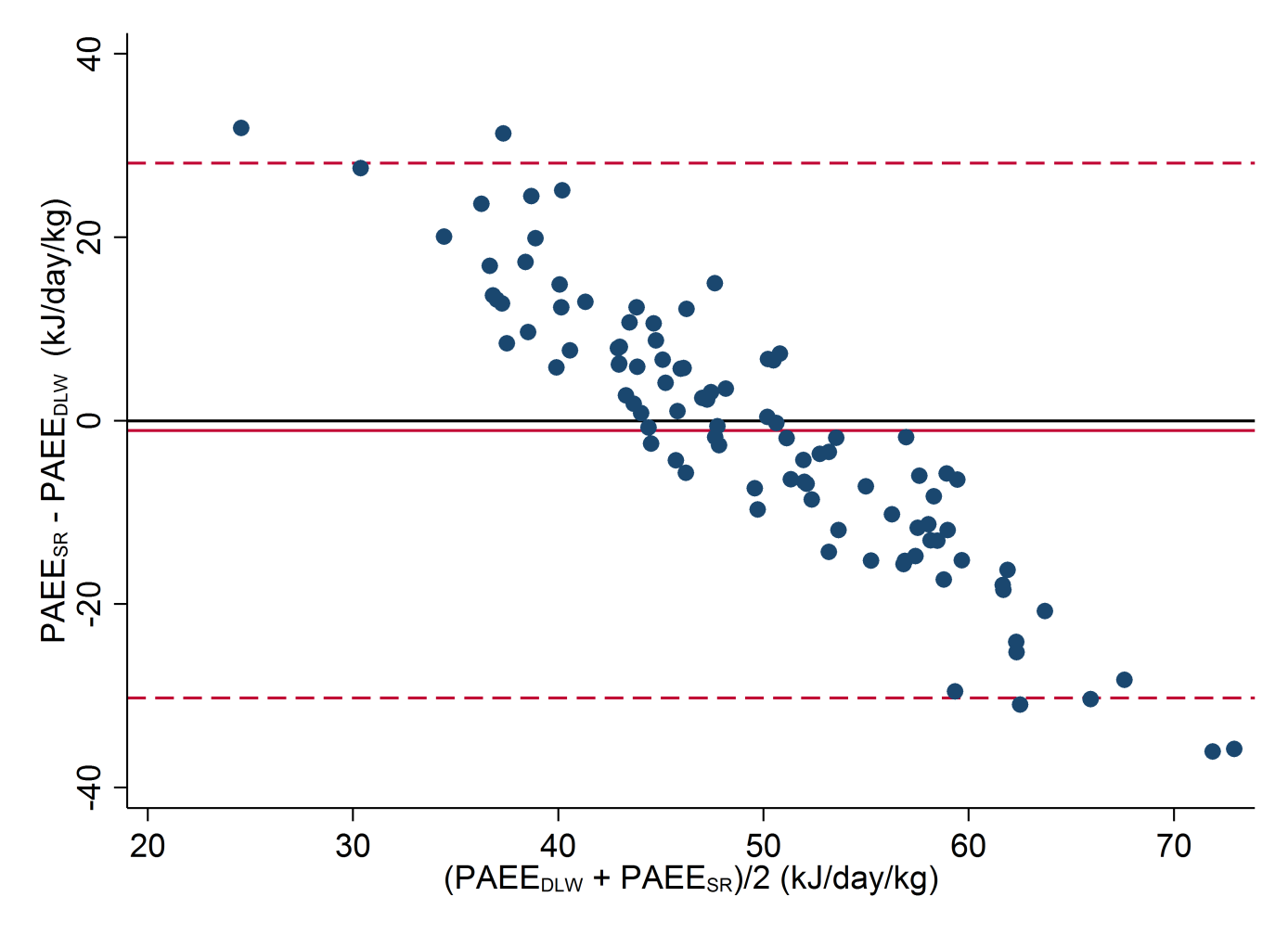** |
| --- |
| **Fig S2.** Differences between physical activity energy expenditure predicted from self-report (PAEE_SR_) and doubly labelled water based PAEE (PAEE_DLW_), plotted against their mean. Reference lines indicate mean difference (dotted) and 95% limits of agreement (dashed). n=98. |

| 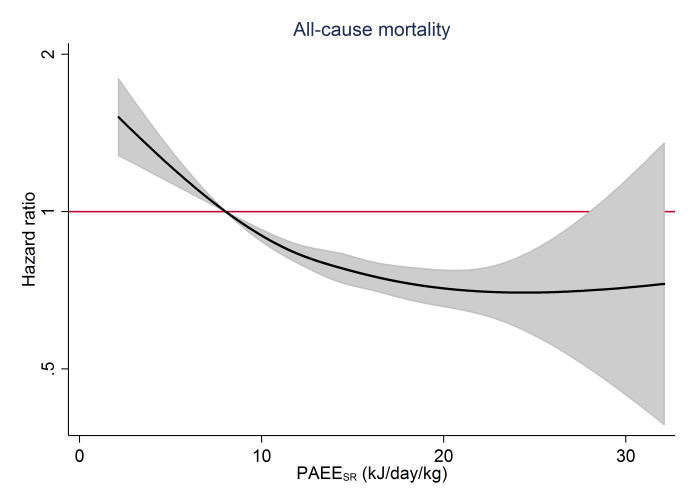 | 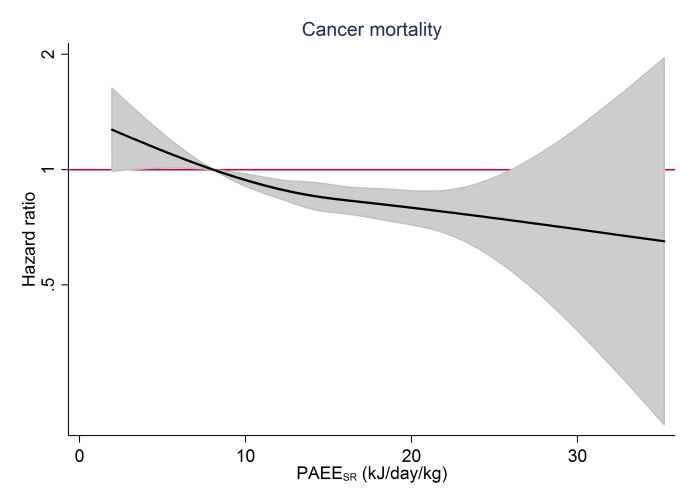 | 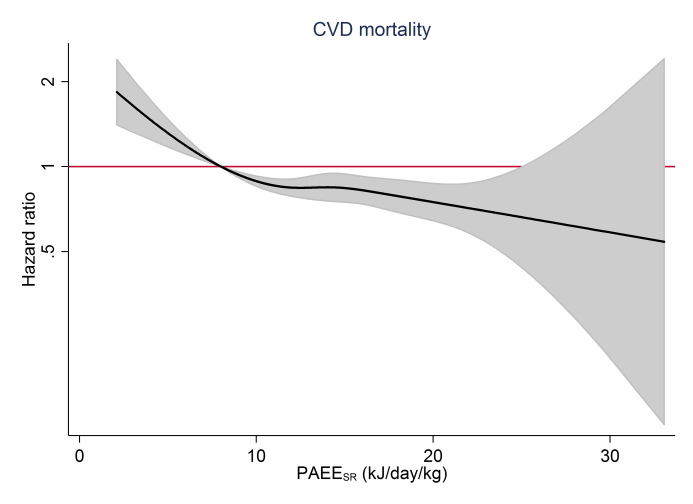 |
| --- | --- | --- |
| 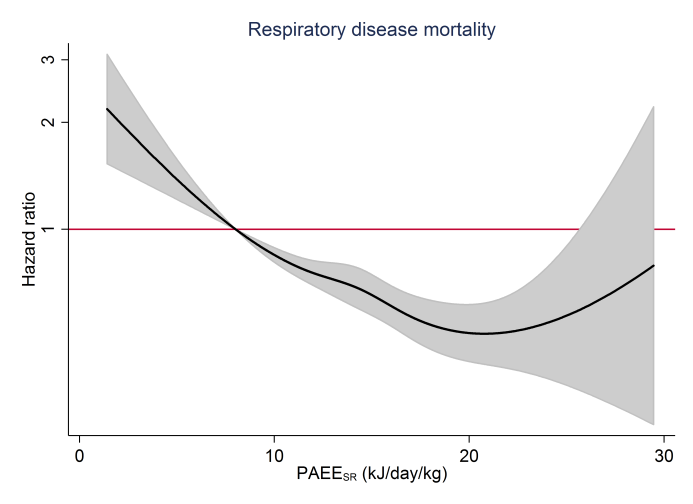 | 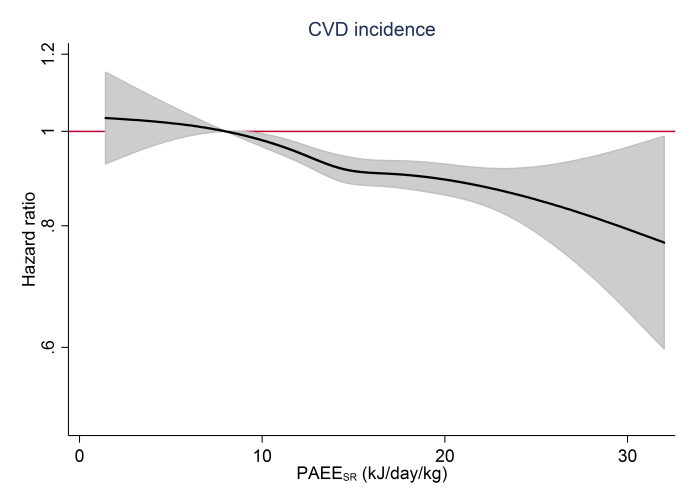 | 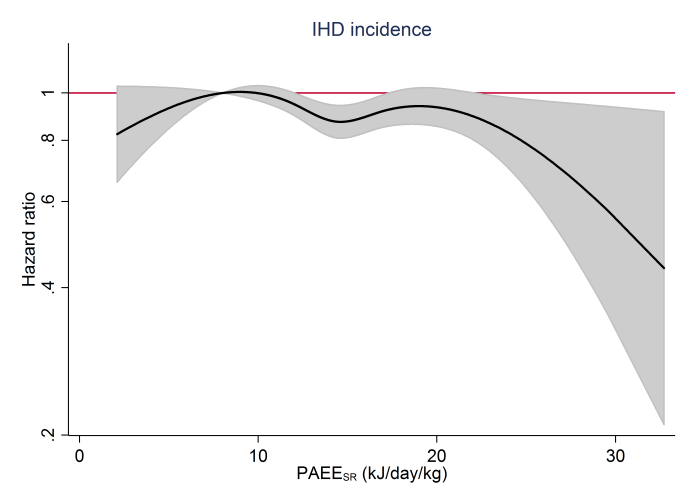 |
| 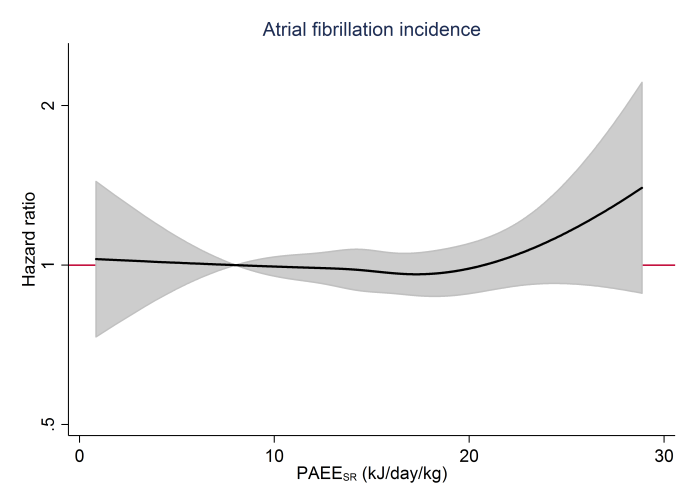 | 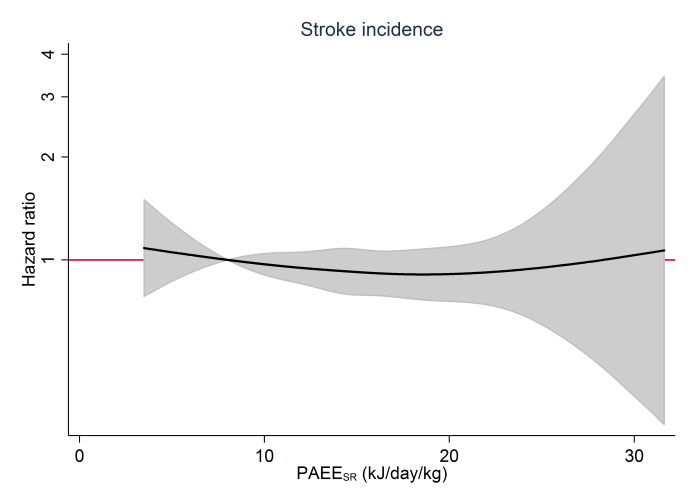 | 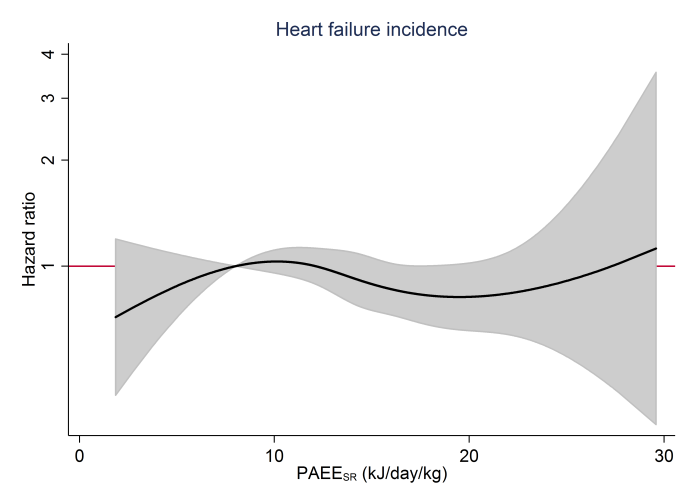 |
| 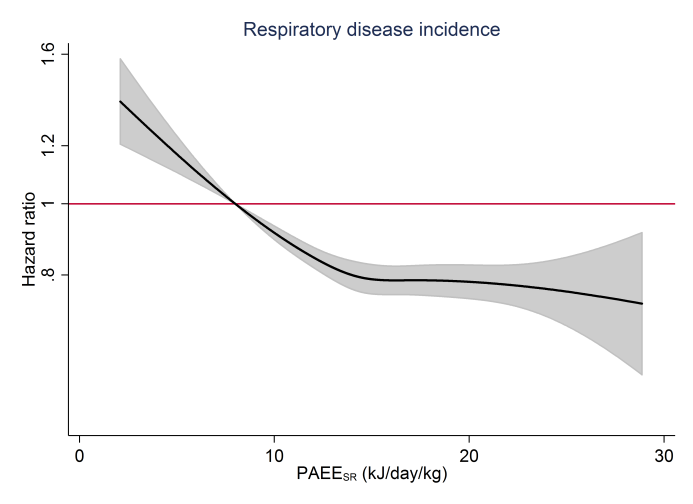 | 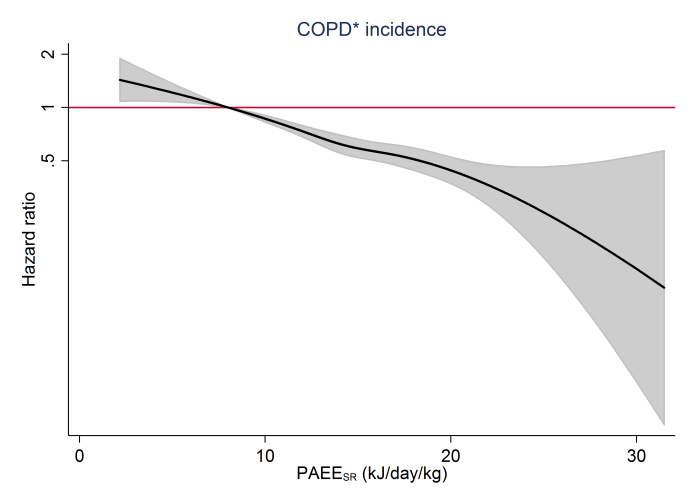 | 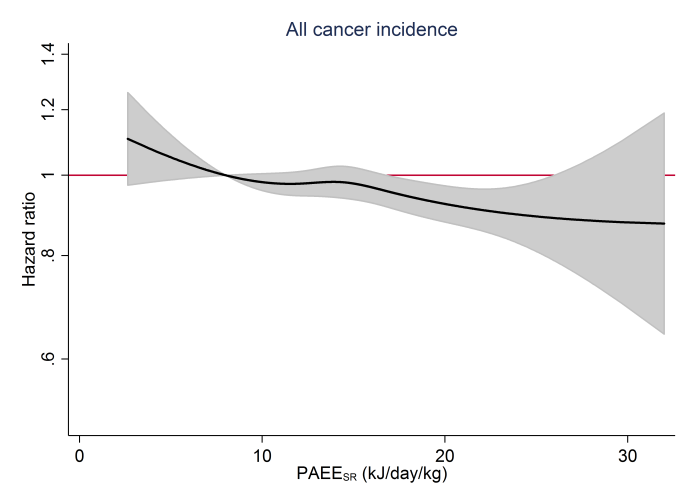 |
| 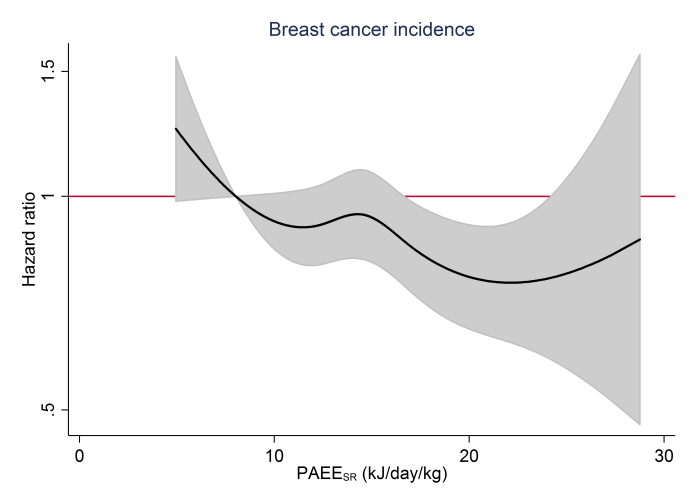 | 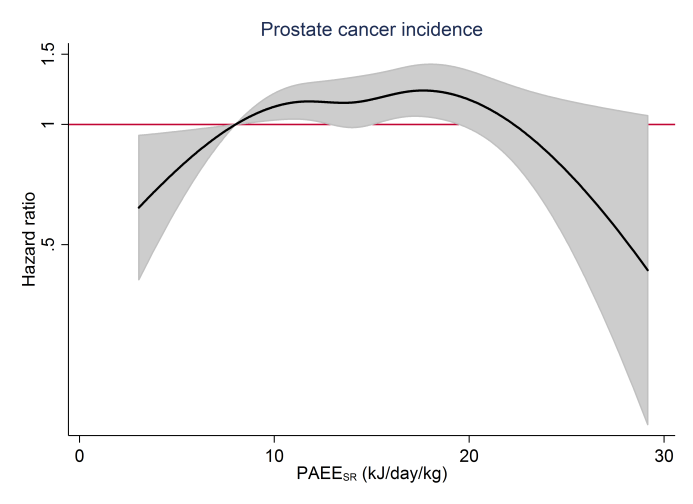 | 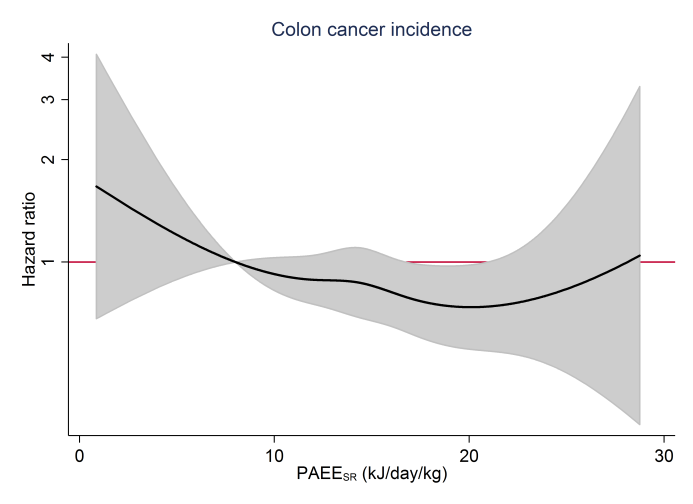 |
| 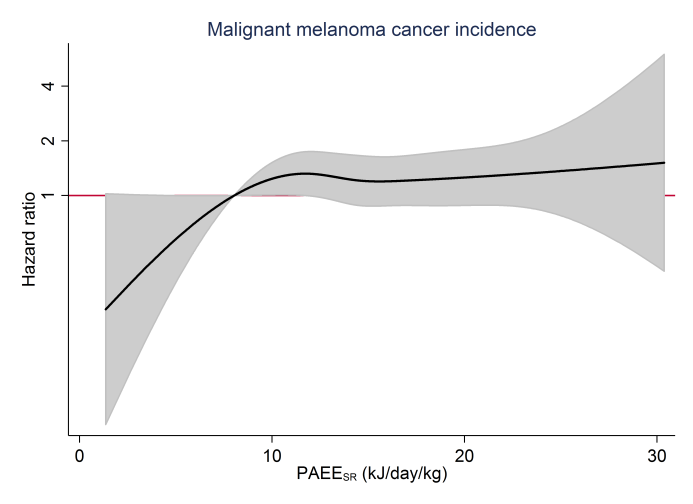 | 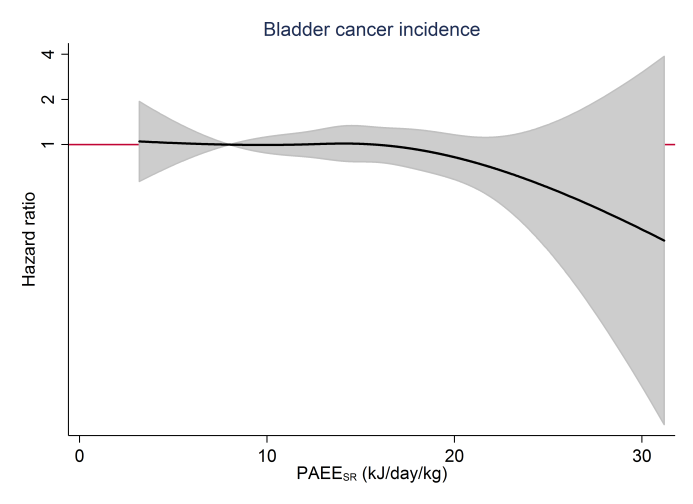 | 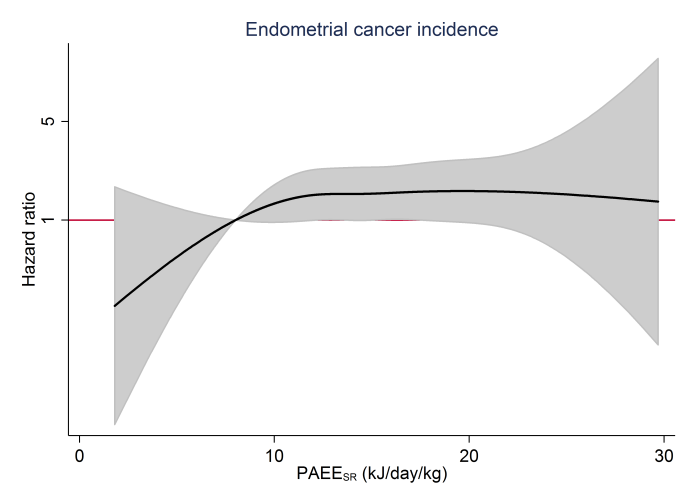 |
| 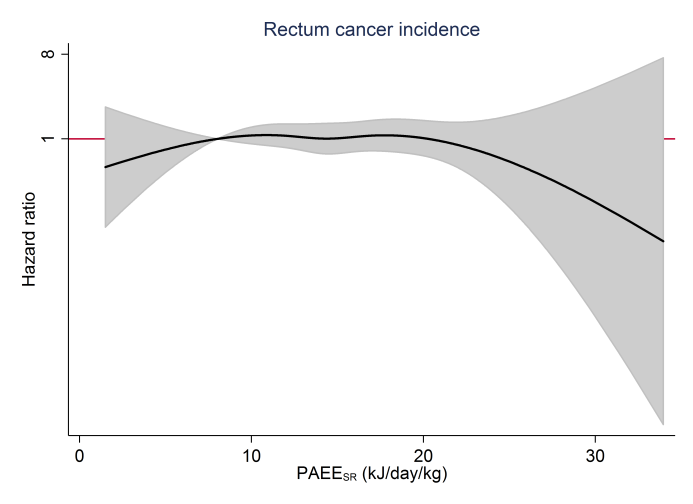 | 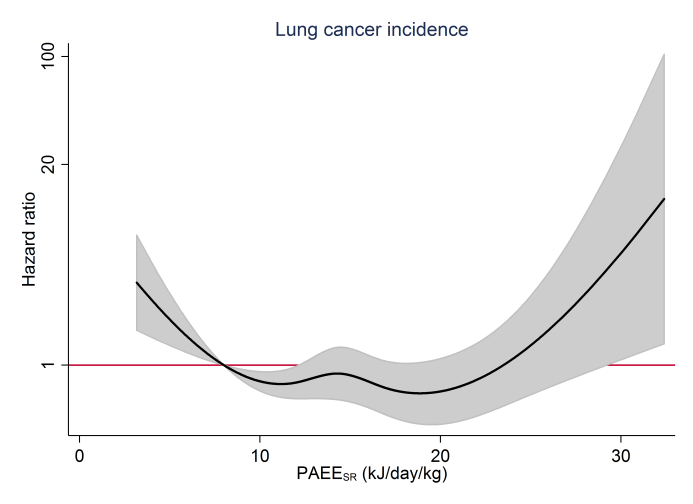 | 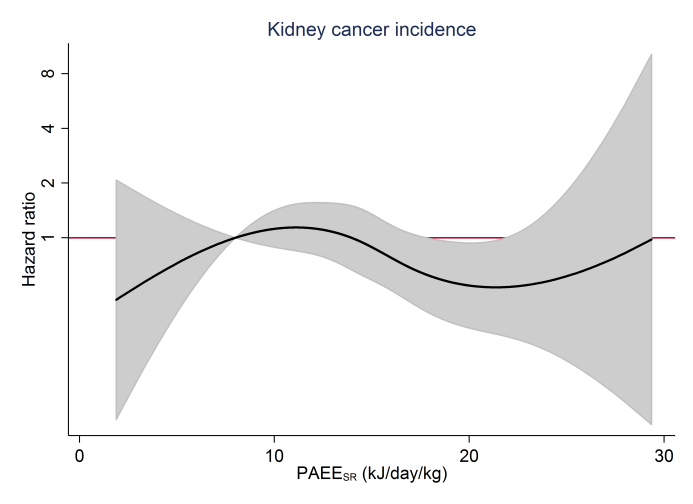 |
| 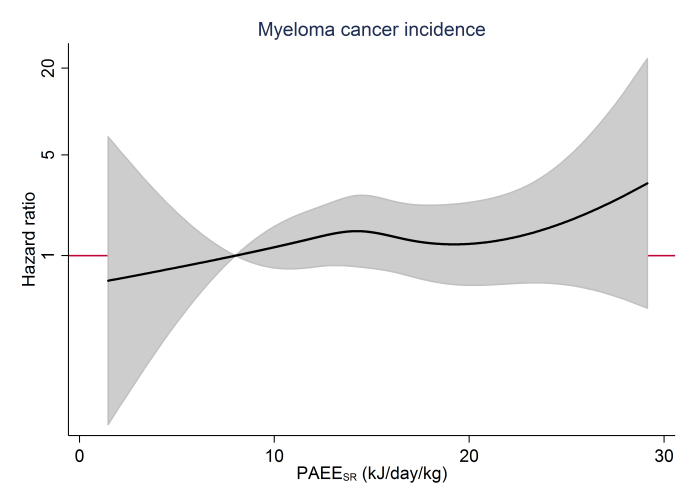 | 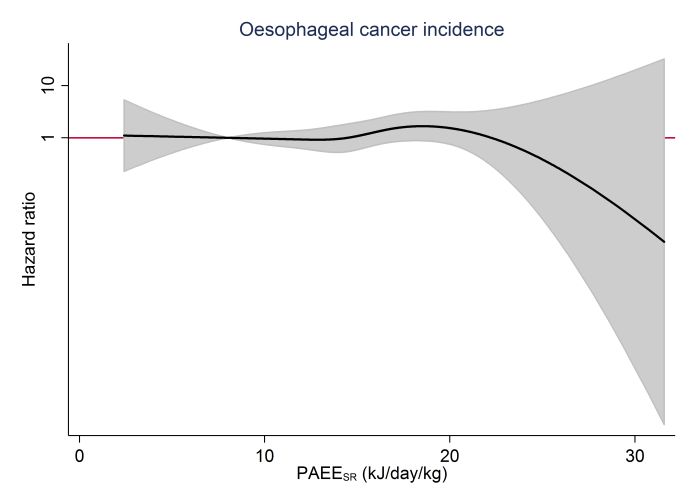 | 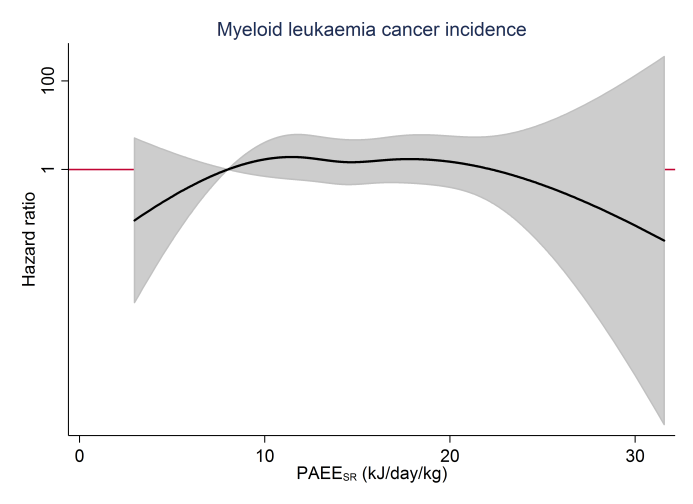 |
| 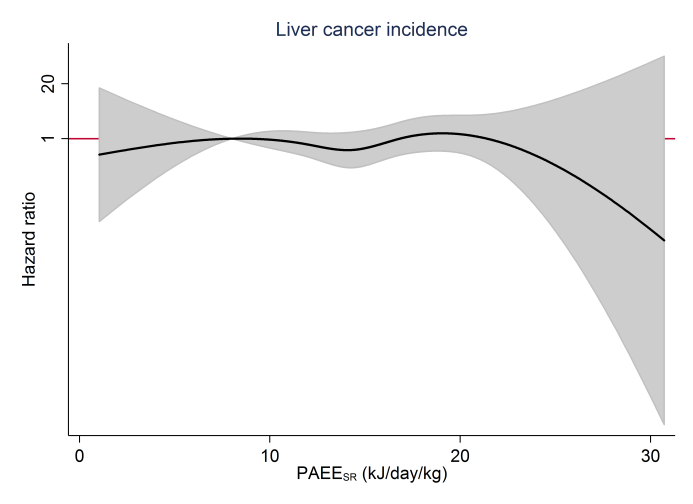 | 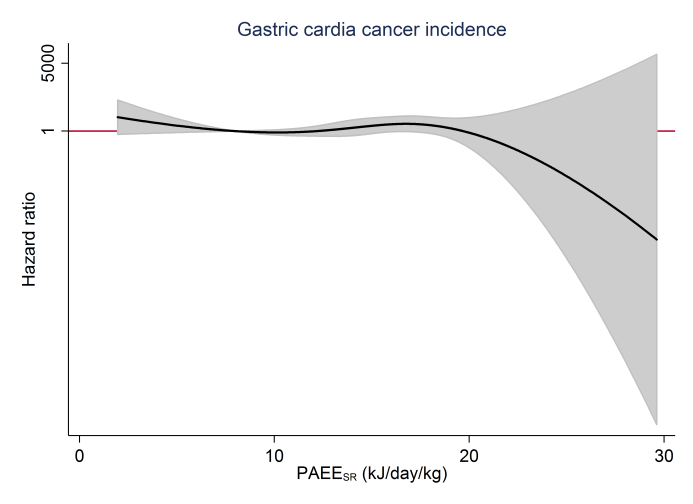 |  |
| **Fig S3.** Hazard ratio and 95% confidence intervals for association between physical activity energy expenditure predicted from self-report (PAEE_SR_) and disease outcomes in UK Biobank. PAEE_SR_ is rescaled to the level of physical activity above the least active participant. Reference HR indicates the level of an individual reporting no leisure-time physical activity, eight hours per day of sedentary occupation, two hours per day of television viewing, two hours per day of computer use, motorised transport for commuting and getting about, and sleeping for ≥9 hours per day.  Adjusted for age (as timescale), sex, ethnicity, Townsend deprivation index (baseline hazard stratification), highest educational level, employment status, alcohol drinking status (baseline hazard stratification), smoking status, salt added to food, oily fish intake, fruit and vegetable intake, processed and red meat intake, body mass index, parental history of cancer, parental history of [heart disease, stroke, hypertension or diabetes], use of blood pressure medication, use of cholesterol lowering medication, doctor-diagnosed diabetes or treatment with insulin.  COPD=chronic obstructive pulmonary disease; CVD=cardiovascular disease; IHD=ischaemic heart disease. | | |

*COPD incidence likely only represents the most severe cases as only approximately 25% of COPD cases are picked up in Hospital Episode Statistics data, compared to national surveys (1).

| **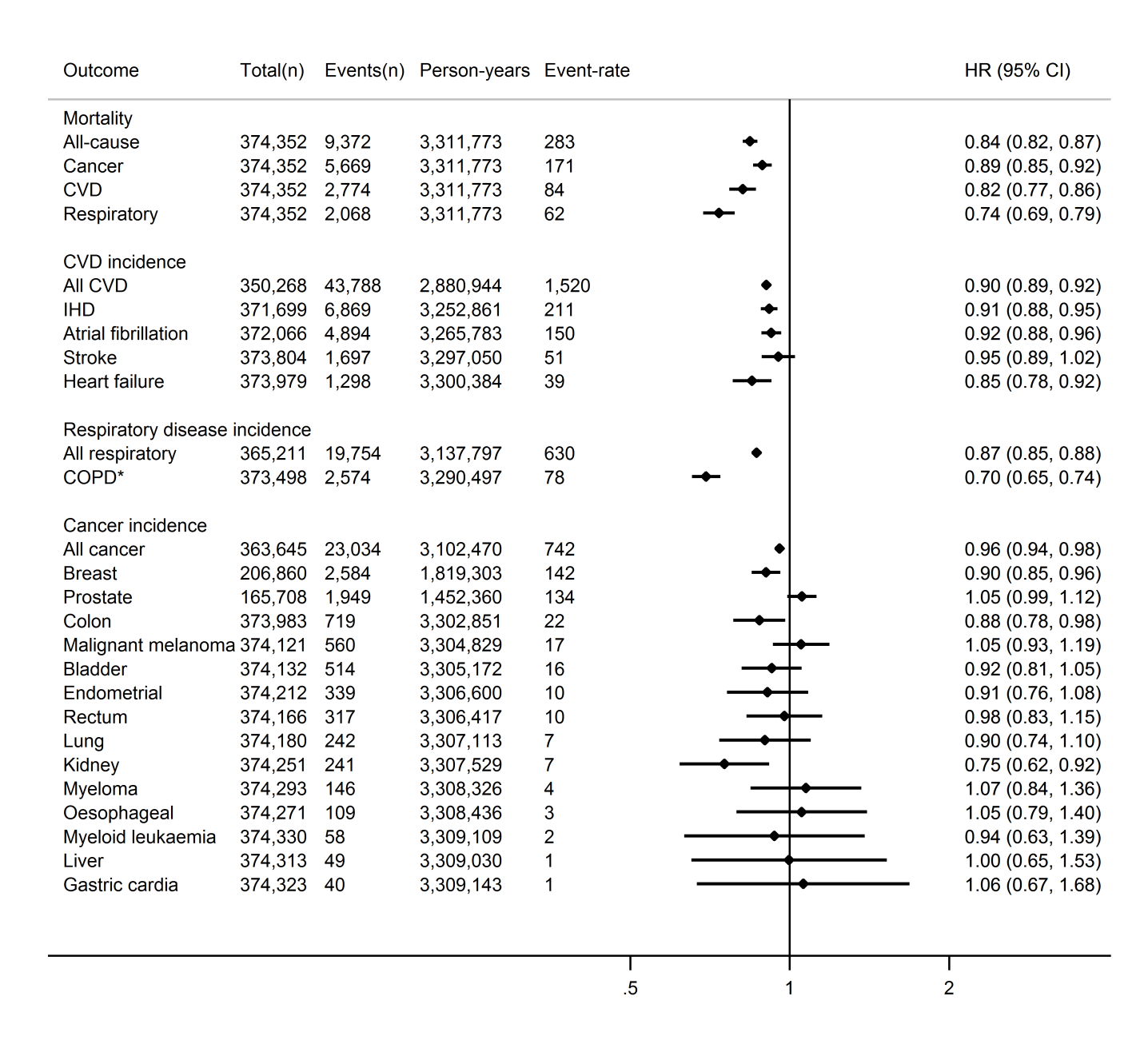** |
| --- |
| **Fig. S4** Hazard ratio (HR) and 95% confidence interval (CI) for linear associations of physical activity energy expenditure predicted from self-report (PAEE_SR_, per 5 kJ/day/kg increments) with fatal and non-fatal outcomes in UK Biobank. Event-rate per 100,000 person years.  Adjusted for age (as timescale), sex, ethnicity, Townsend deprivation index (baseline hazard stratification), highest educational level, employment status, alcohol drinking status (baseline hazard stratification), smoking status, salt added to food, oily fish intake, fruit and vegetable intake, processed and red meat intake, parental history of cancer, parental history of [heart disease, stroke, hypertension or diabetes], use of blood pressure medication, use of cholesterol lowering medication, doctor-diagnosed diabetes or treatment with insulin.  COPD=chronic obstructive pulmonary disease; CVD=cardiovascular disease; IHD=ischaemic heart disease.  *COPD incidence likely only represents the most severe cases as only approximately 25% of COPD cases are picked up in Hospital Episode Statistics data, compared to national surveys (1). |
|  |
|  |

| **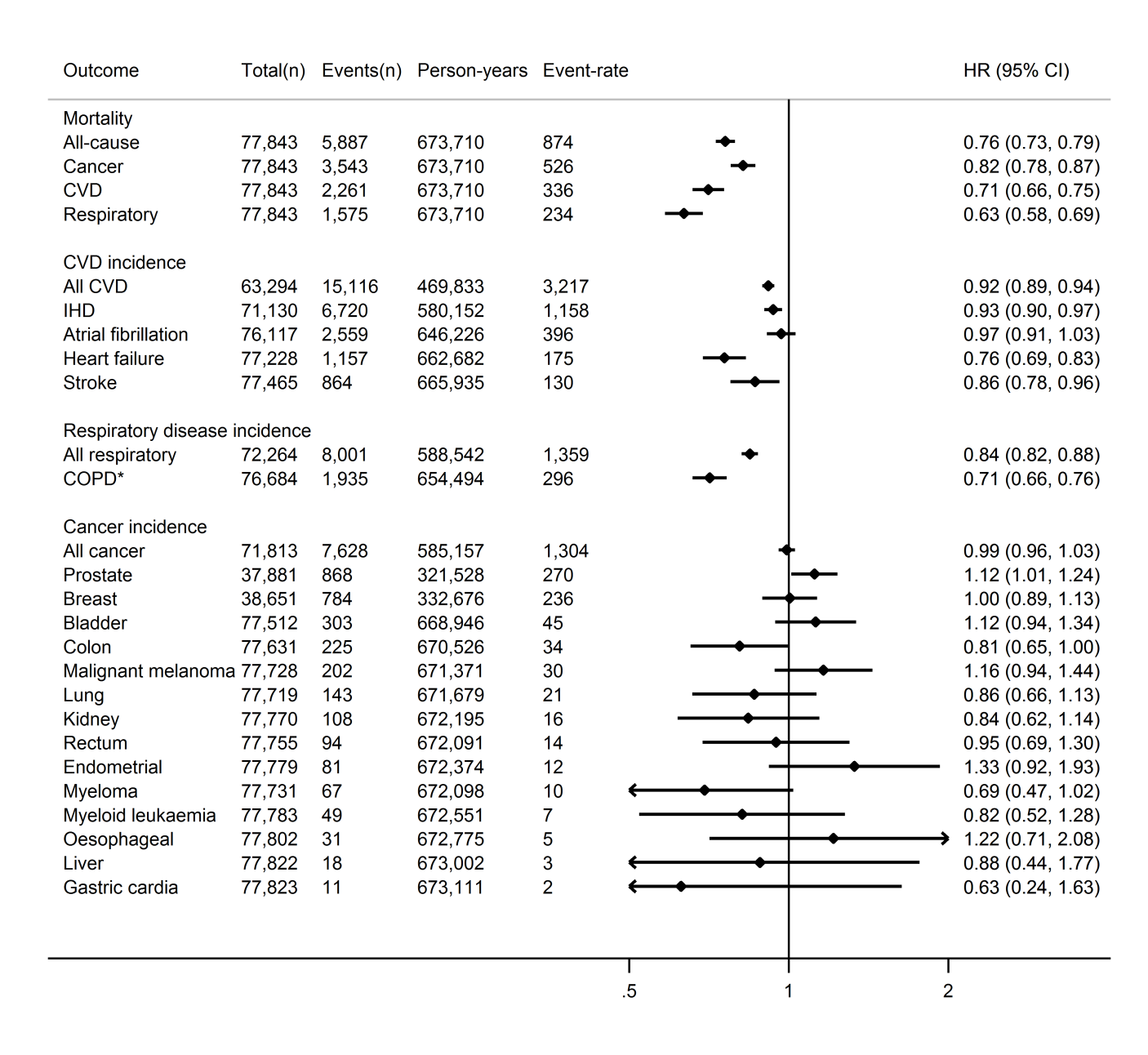** |
| --- |
| **Fig. S5** Hazard ratio (HR) and 95% confidence interval (CI) for linear associations of physical activity energy expenditure predicted from self-report (PAEE_SR_, per 5 kJ/day/kg increments) with fatal and non-fatal outcomes in UK Biobank. Event-rate per 100,000 person years.  Adjusted for age (as timescale), sex, ethnicity, Townsend deprivation index (baseline hazard stratification), highest educational level, employment status, alcohol drinking status (baseline hazard stratification), smoking status, salt added to food, oily fish intake, fruit and vegetable intake, processed and red meat intake, body mass index, parental history of cancer, parental history of [heart disease, stroke, hypertension or diabetes], use of blood pressure medication, use of cholesterol lowering medication, doctor-diagnosed diabetes or treatment with insulin.  COPD=chronic obstructive pulmonary disease; CVD=cardiovascular disease; IHD=ischaemic heart disease.  *COPD incidence likely only represents the most severe cases as only approximately 25% of COPD cases are picked up in Hospital Episode Statistics data, compared to national surveys (1). |
|  |
|  |

**Reference**

(1) Rothnie, K. J., Su, B., Newson, R., Quint, J. K. & Soljak, M. *COPD prevalence model for small populations: Technical Document produced for Public Health England*. (2019).
